# Sensitivity Analysis of a Transmission Interruption Model for the Soil-Transmitted Helminth Infections in Kenya

**DOI:** 10.3389/fpubh.2022.841883

**Published:** 2022-03-25

**Authors:** Collins Okoyo, Nelson Onyango, Idah Orowe, Charles Mwandawiro, Graham Medley

**Affiliations:** ^1^Eastern and Southern Africa Centre of International Parasite Control (ESACIPAC), Kenya Medical Research Institute (KEMRI), Nairobi, Kenya; ^2^School of Mathematics, University of Nairobi, Nairobi, Kenya; ^3^Faculty of Public Health and Policy, London School of Hygiene and Tropical Medicine (LSHTM), London, United Kingdom

**Keywords:** mathematical model, soil-transmitted helminths, sensitivity analysis, extended Fourier Amplitude Sensitivity Test, Kenya

## Abstract

As the world rallies toward the endgame of soil-transmitted helminths (STH) elimination by the year 2030, there is a need for efficient and robust mathematical models that would enable STH programme managers to target the scarce resources and interventions, increase treatment coverage among specific sub-groups of the population, and develop reliable surveillance systems that meet sensitivity and specificity requirements for the endgame of STH elimination. However, the considerable complexities often associated with STH-transmission models underpin the need for specifying a large number of parameters and inputs, which are often available with considerable degree of uncertainty. Additionally, the model may behave counter-intuitive especially when there are non-linearities in multiple input-output relationships. In this study, we performed a global sensitivity analysis (GSA), based on a variance decomposition method: extended Fourier Amplitude Sensitivity Test (eFAST), to a recently developed STH-transmission model in Kenya (an STH endemic country) to; (1) robustly compute sensitivity index (SI) for each parameter, (2) rank the parameters in order of their importance (from most to least influential), and (3) quantify the influence of each parameter, singly and cumulatively, on the model output. The sensitivity analysis (SA) results demonstrated that the model outcome (STH worm burden elimination in the human host) was significantly sensitive to some key parameter groupings: combined effect of improved water source and sanitation (ϕ), rounds of treatment offered (τ), efficacy of the drug used during treatment (*h*), proportion of the adult population treated (*g*_*a*_: akin to community-wide treatment), mortality rate of the mature worms in the human host (μ), and the strength of the -dependence of worm egg production (γ). For STH control programmes to effectively reach the endgame (STH elimination in the entire community), these key parameter groupings need to be targeted since together they contribute to a strategic public health intervention.

## 1. Introduction

Mathematical models are nowadays gaining increasing popularity in the study of the dynamics of infectious diseases, particularly to examine, explain and predict the infection transmission and eventual elimination (1–3). Over the years, specific models have been developed for specific diseases of global importance with the overarching aim of developing public health strategies for control, prevention and elimination (1, 3–5). These models provide a mathematical representation of the underlying dynamics of the infection transmission cycle that usually involve complex interactions between infected individuals, susceptible hosts and the infectious materials, and this dynamic is generally expressed as a set of dynamical ordinary differential equations (ODEs) (5). Model outputs, which are usually the ODE solutions over a simulation interval, provide a dynamic representation of the transmission process (6). Parameters used in the computation of these models are normally estimated from observational or experimental data, and in cases where these parameter values are unavailable, they are often set to plausible value ranges based on literature reviews, analogous systems, statistical inferences, or experts opinion (7). However, most model outputs often have complex, nonlinear relationships with the model parameters, hence inappropriate parameter value choices coupled with parameter uncertainty can lead to bias in model outputs (3, 8).

The study of how the uncertainty in the output of a mathematical model or system (numerical or otherwise) might be divided and assigned to various sources of uncertainty in its parameters is known as sensitivity analysis (SA) (3, 9). SA is a powerful tool for studying and understanding the underlying behavior of a numerical model, additionally, it allows the quantification of the sensitivity in the model outputs to changes in each of the model parameters. Methods for performing SA can be broadly classified into two; (1) local sensitivity analysis (LSA) methods, which imply that the inputs are varied one at a time by a small amount around some fixed point and the effect of individual perturbations on the output are calculated, and (2) global sensitivity analysis (GSA) methods, where all inputs are varied simultaneously over their entire input space, typically using a sampling-based approach, and the effects on the output of both individual inputs and interactions between inputs are assessed (10, 11). Whereas LSA methods are easier to implement and deemed useful in some situations, they however lack some essential desired properties as described by Andrea and colleagues (12). GSA techniques are today becoming increasingly more common since they explore the entire input space (or the full spectrum of each factor), hence their results do not depend on the central values (13). For this reason, GSA methods possess multidimensional scaling property (12). GSA methods include; variance-based methods (e.g., Sobol' and Fourier Amplitude Sensitivity Test (FAST)) (14, 15), global screening methods (e.g., Morris method which is also called the elementary effect method) (16), sampling-based methods (e.g., Monte Carlo filtering and Latin hypercube sampling with partial rank correlation coefficient (LHS-PRCC) index) (17, 18), and the recently developed sensitivity heat map (SHM) method (19), and among other methods.

Whilst GSA techniques have been broadly applied to various mathematical models in the areas of systems biology modeling (20), environmental modeling (21, 22), and infectious diseases modeling (3, 23–25), however, these techniques have not been specifically applied to models studying the transmission of soil-transmitted helminths (STH) infections, especially with regards to Kenya infection transmission setting. Briefly, STH are part of a group of diseases categorized as neglected tropical diseases (NTDs) (26). Currently, the global burden of STH is estimated at 1.9 million disability-adjusted life years (DALYs) (27), but with up to four billion people estimated to be at risk (28). STH are the most prevalent group of intestinal helminths (26), and they are mainly transmitted through ingestion of nematode eggs from contaminated soil (for the case of *Ascaris lumbricoides* or *Trichuris trichiura*) or active penetration of the skin by larvae in the soil (for the case of hookworms: *Necator americanus* and *Ancylostoma duodenale*) (29). STH can be controlled through mass treatment with either albendazole, mebendazole, levamisole or pyrantel drugs (30), and the treatment impact can be sustained with a revamped complementary water, sanitation and hygiene (WASH) interventions (31).

Recently, interest has increased in the use of mathematical models to determine the STH transmission dynamics (4, 32), infection transmission interruption (33–36), and different interventions impact (36). These mathematical models serve an important role in guiding the design and implementation of epidemiological studies and public health policy formulation (37). STH models have incorporated variety of modeling approaches including both deterministic (33, 36), and individual-based stochastic simulation (32, 38). Even though progress has been made in model formulation, parameter estimation and application, most of these models have estimated their parameters theoretically with little use of existing data coupled with considerable degree of uncertainty, and with less exploration of sensitivity analysis to determine the most important parameters and their usefulness in influencing the interventions impact.

Estimation of epidemiological parameters like infection transmission rate, relative contribution of infectious materials to the environment by the host and the average number of new parasite offsprings caused by one typical parasite (simply, the reproduction number; *R*_*o*_), is an essential task when analyzing STH transmission models (33). However, in some situations, some parameters of interest may not be estimated directly from the available data (39). Hence, an indirect approach may be adopted in which a mathematical model of the transmission process is formulated and fitted to the data (40). In the process, the parameters estimated may have uncertainty due to noise in the data, but this will depend on the parameter estimation approach chosen for the model (41). Standard statistical approaches can be used to quantify the uncertainty in parameter estimates that emanate from the noise in the data (42). However, these approaches may not sufficiently provide insight into the sensitivity of the estimates to the model output (43). Therefore, sensitivity analysis is desired when model-based approaches are used to interpret epidemiological data (3).

To the best of our knowledge advanced sensitivity analysis techniques involving STH transmission models has been rarely investigated and adopted. This study will add to the body of knowledge on application of sensitivity analysis methods to the broader category of infectious disease models. The main objective of this article was therefore to investigate and apply a robust global sensitivity analysis method: the *extended Fourier Amplitude Sensitivity Test (eFAST)*, a global variance decomposition based method, to STH transmission model previously developed by Okoyo and colleagues (36), in order to quantify the uncertainty in the parameters estimated and to determine the most useful parameters to the model output. Our sensitivity analysis process fully used the available data from the Kenyan STH transmission setting. These results are significant to the Kenyan STH control program and to the global STH community, especially at this time when control programs are aiming to eliminate STH by the year 2030 (44, 45).

## 2. Materials and Methods

### 2.1. Data and Data Sources

Since the year 2012, Kenya has been conducting a nation-wide deworming program mainly among the pre-school and school-aged children (46). This program targeted to reduce the prevalence of STH infections to below 1%, and subsequently to interrupt the infection transmission as per the world health organization (WHO) guidelines (47) and targets (44). The data for monitoring and evaluation (M&E) to assess the program impact is independently collected by the Kenya Medical Research Institute (KEMRI). Therefore, in this analysis, we used the five-year (2012-2017) data on STH infections collected by the M&E team of the deworming program (48). Key indicators collected included type of worm, number of eggs observed in each individual surveyed, rounds of treatment administered, treatment coverage, and information on WASH conditions. Other model parameter values were based on previous studies conducted in Kenya.

### 2.2. Model Specification

In this study, we are referring to a previously formulated and analyzed STH transmission interruption model in Kenya by Okoyo and colleagues (36). Briefly, this model studied the dynamics of STH transmission and elimination in three age groups; pre-school aged children (PSAC: 2–4 years), school aged children (SAC: 5–14 years), and adults (above 14 years) as well as the dynamics of infectious materials in the environment. It sought to determine the effect of two interventions, mass drug administration (MDA) and WASH, and the projected STH elimination period considering the impact of each of the these two interventions.

We consider the below model from the previous work (36),


(1) $$\begin{array}{r} \begin{array}{r} \frac{dM_{p}}{dt}=\beta_{p}(1-\phi )L-(\mu +c_{p})M_{p}\\ \frac{dM_{c}}{dt}=\beta_{c}(1-\phi )L-(\mu +c_{c})M_{c}\\ \frac{dM_{a}}{dt}=\beta_{a}(1-\phi )L-(\mu +c_{a})M_{a}\\ \frac{dL}{dt}=\left[(1-\phi )\sum_{i}f(M_{i};k,\gamma )n_{i}\lambda_{i}\right]-\\ \left[\mu_{L}+(1-\phi )\sum_{i}\beta_{i}n_{i}\right]L;\text{ for }i=p,c,a \end{array}\} \end{array}$$

Where, *M*_*p*_, *M*_*c*_, and *M*_*a*_ are the mean worm burdens in the three age groups (PSAC, SAC and adults) respectively; *L* is the per capita infectiousness of the shared reservoir; β_*i*_ (for *i* = *p, c, a*) is the strength of infectious contact with the reservoir for each age group, respectively (i.e., the transmission rate); μ is the mortality rate of the mature worms in the hosts; λ_*i*_ (for *i* = *p, c, a*) describes the relative per capita contributions of infectious materials by each age group (i.e., the contamination rate); *n*_*i*_ (for *i* = *p, c, a*) is the proportion of the population in each age group; μ_*L*_ is the rate of decay of infectious materials in the environment; and ϕ is the simulated combined effect of improved water source and sanitation (i.e., WASH) at individual level. The treatment effect illustrating the impact of MDA on the mean worm burden and egg production output for each age group was denoted as,


(2) $$\begin{array}{l} c_{i}=\frac{-ln\left(1-g_{i}h\right)}{\tau };\text{for }i=p,c,a \end{array}$$

where *g*_*i*_ (for *i* = *p, c, a*) denotes the proportion of individuals treated in each age group per treatment round, *h* the drug efficacy and τ the interval between the treatment rounds. Additionally, the function,


(3) $$\begin{array}{l} f\left(M_{i};k,\gamma \right)=\frac{M_{i}}{{\left[1+\frac{M_{i}}{k}\left(1-e^{-\gamma }\right)\right]}^{k+1}};\text{for }i=p,c,a \end{array}$$

describes the mean egg production rate from each age group with the mean worm burden (*M*_*i*_) which assumed a negative binomial distribution with aggregation parameter (*k*), and the resulting impact of the density-dependence of egg production from each host's worm burden described by the parameter, *exp*(−γ).

Further details about this model's Equation (1) description, formulation and analysis are provided in the paper by Okoyo and colleagues (36). In the current study, we aimed to perform sensitivity analysis on this model's output for each of the parameters described in Table 1. To the best of our knowledge, this is the first application of a global sensitivity analysis method to a fully age structured STH transmission model of this nature (ODE model). Specifically, we performed sensitivity analysis using a robust and efficient global sensitivity analysis method to compute the first and total-order sensitivity indices of each parameter to enable us rank the influence and quantify the significant effect of each parameter to the model outcome (worm burden elimination). This kind of analysis is important in enabling STH control programmes to target the scarce resources to the most influencing parameters that would directly impact on reducing the host's worm burden, hence facilitating an efficient way of reaching STH elimination.

**Table 1:** Input parameters for the analyzed model.

**No**.	**Notation**	**Definition**	**Assumed distribution**	**Input value range (min, max)**	**Measurement**
1.	β_*p*_	Infection transmission rate among PSAC	Uniform	(0.455, 1.82)	Rate
2.	β_*c*_	Infection transmission rate among SAC	Uniform	(0.49, 1.96)	Rate
3.	β_*a*_	Infection transmission rate among adults	Uniform	(0.385, 1.54)	Rate
4.	μ	Mortality rate of the mature worm in the host	Uniform	(0.5, 2.0)	Years
5.	μ_*L*_	Mortality rate of the free-living infectious materials in environment	Uniform	(0.115, 0.46)	Years
6.	*n*_*p*_	Proportion of PSAC in the population	Uniform	(0.025, 0.1)	Population proportion
7.	*n*_*c*_	Proportion of SAC in the population	Uniform	(0.125, 0.5)	Population proportion
8.	*n*_*a*_	Proportion of adults in the population	Uniform	(0.35, 1.4)	Population proportion
9.	λ_*p*_	Relative contributions of PSAC to the environment	Uniform	(1.25, 5.0)	Rate
10.	λ_*c*_	Relative contributions of SAC to the environment	Uniform	(2.0, 8.0)	Rate
11.	λ_*a*_	Relative contributions of adults to the environment	Uniform	(1.75, 7.0)	Rate
12.	*k*	Over-dispersion (aggregation) parameter	Uniform	(0.285, 1.14)	Rate
13.	γ	Strength of density dependence of worm egg production	Uniform	(0.00175, 0.007)	Rate
14.	ϕ	Combined improved water and sanitation (WASH) effect	Uniform	(0.25, 1.0)	Coverage proportion
15.	τ	Interval between treatment rounds per year	Uniform	(0.25, 1.0)	Treatment rounds
16.	*g*_*p*_	Proportion of PSAC treated	Uniform	(0.25, 1.0)	Treatment proportion
17.	*g*_*c*_	Proportion of SAC treated	Uniform	(0.25, 1.0)	Treatment proportion
18.	*g*_*a*_	Proportion of adults treated	Uniform	(0.25, 1.0)	Treatment proportion
19.	*h*	Drug efficacy	Uniform	(0.25, 1.0)	Efficacy level

*The references for the initial values were provided during the original model formulation and analysis (36)*.

### 2.3. Overview of Sensitivity Analysis Methods

SA methodological approaches can be divided into three categories based on their methodology: (1) mathematical; (2) statistical; and (3) graphical (12, 49). The classification of SA methodology aids in determining whether or not a method is appropriate for a certain model and analysis goal. Table 2 summarizes some of the commonly used sensitivity analysis methods.

**Table 2:** A summary of key sensitivity analysis methods, stating whether they are local or global methods, their ability to detect interactions between inputs and handle nonlinearities in the model, their computational cost, number of parameter evaluations and aims.

**Methods**	**Type**	**Interactions**	**Nonlinearities**	**Computational cost**	**Evaluations**	**Aim(s)**
One-way	Local	No	No	Low	OAT	Rank
Multi-way	Local	No	No	Low	MAT	Rank
Local-derivative	Local	No	No	Low	OAT	Rank
Morris	Global	Yes	Yes	Medium	OAT	Screen
Sobol	Global	Yes	Yes	High	MAT	Rank, Screen
FAST/eFAST	Global	Yes	Yes	High	MAT	Rank, Screen
DGSM	Global	Yes	Yes	Medium	OAT	Screen
Sensitivity index	Local	No	No	Low	OAT	Rank
Importance index	Global	No	Yes	Low	OAT	Rank
*CC*_*i*_	Global	No	No	Medium	OAT	Rank
*SRC*_*i*_	Global	No	No	Medium	OAT	Rank
*PRC*_*i*_	Global	Yes	No	Medium	OAT	Rank
*SRCC*_*i*_	Global	No	Yes	Medium	OAT	Rank
*PRCC*_*i*_	Global	Yes	Yes	Medium	OAT	Rank

*The table was adopted from a sensitivity analysis methods review paper (57)*.

*FAST: Fourier Amplitude Sensitivity Test; eFAST: extended Fourier Amplitude Sensitivity Test*.

*DGSM: Derivative-based global sensitivity measures; CC_i_: Pearson's correlation coefficient; SRC_i_: Standardized regression coefficient*.

*PRC_i_: Partial regression coefficient; SRCC_i_: Spearman rank correlation coefficient; PRCC_i_: Partial rank correlation coefficient*.

*OAT: One-at-a-time; MAT: Many-at-a-time*.

Mathematical methods are used to determine how sensitive a model's output is to a given input's range of fluctuation. These methods usually entail computing the output for a few input values that indicate the input's probable range. These methods do not account for variance in the output owing to variance in the inputs, but they can be used to examine the impact of a wide range of input values on the output (50). Mathematical approaches can aid with input screening, verification and validation, as well as identifying inputs that require additional data or investigation (51). Mathematical methods include nominal range SA, break-even analysis, difference in log-odds ratio, automatic difference, and among others.

Statistical approaches entail running simulations with probability distributions allocated to the inputs and analyzing the impact of variation in the inputs on the output distribution (52). One or more inputs are varied at a time, depending on the method. Statistical approaches can be used to determine the effect of interactions among numerous inputs. Various strategies, such as Monte Carlo simulation, Latin hypercube sampling, and other methods, can be used to determine the range and relative likelihood of inputs (53). A variety of strategies can be used to assess the model's sensitivity to individual or groups of inputs (52). Statistical approaches include regression analysis, analysis of variance, response surface methods, FAST method, mutual information index, and among others.

Graphical approaches illustrate sensitivity using graphs, charts, or surfaces. In general, graphical tools are used to show how variations in inputs affect an output (54). Graphical approaches can be used to screen a model before further investigation or to illustrate complex input–output dependencies (55). For a better depiction, graphical methods can be utilized to supplement the results of mathematical and statistical methods (56). Graphical methods include scatter plots, cobweb plots, contribution to the sample mean (CSM) plots, and among others.

### 2.4. Sensitivity Analysis Using eFAST Method

The *Fourier Amplitude Sensitivity Test* is a global sensitivity analysis method based on the variance decomposition technique, it was developed by Cukier and colleagues in the early 1970s (58). This GSA method uses sinusoidal functions (*x* = *f*(*N*_*s*_)) and the orthogonality property of the Fourier Series (FS) (59) to give an approximation of the total model variance in terms of the real and imaginary coefficients of the FS (57). First-order index of a particular factor, say *X*_*i*_, is given by the proportion of this total variance attributable to the FS harmonics caused by that particular factor (*X*_*i*_). The first numerical implementation of this calculation was done in the early 1980s by McRae and colleagues (60). However, the major limitation of the FAST approach, is that, it can only compute the first-order indices of each factor, not the total order indices.

Saltelli and colleagues (15), extended the FAST method to include the computation of the total-order indices of each parameter, giving rise to the *extended FAST (eFAST)* method. The eFAST gives quantitative information contained in the first and total-order sensitivity indices (SI). This method is more efficient than the other variance-based methods, like Sobol method, since it calculates all indices in one set of model evaluations (61). However, just like most variance-based methods, eFAST is more computationally expensive than the derivative and regression-based methods.

#### 2.4.1. Computation of the First-Order Sensitivity Index (*SI*_*i*_)

In FAST/eFAST method, input parameters are varied to bring about variation in model output, this variation is quantified using the standard statistical notion of variance;


(4) $$\begin{array}{l} s^{2}=\frac{\sum_{i=1}^{N}{\left(y_{i}-ȳ\right)}^{2}}{N-1} \end{array}$$

where, *N* is the sample size (equivalently, the number of model runs), *y*_*i*_ the *i*^*th*^ model output, and ȳ the sample mean.

The algorithm then partitions the output variance, allocating fractions of the variance explained by variation in each input parameter (i.e., partial variation). Allocation of variation in FAST/eFAST is achieved by varying different parameters at different frequencies, while encoding the identity of parameters in the frequency of their variation (61). The strength of each parameter's frequency in the model output is then measured using Fourier analysis. Thus, how strongly a parameter's frequency propagates from input to the output serves as the measure of the model's sensitivity to the parameter.

Mathematically, we consider the function,


(5) $$\begin{array}{l} Y=f\left(X\right)=f\left(X_{1},X_{2},...,X_{n}\right) \end{array}$$

where *X*_*i*_ = [0, 1]; for *i* = 1, 2, …, *n*.

The key aim of FAST method is to apply the Ergodic theorem (62) to transform the n-dimensional integral


(6) $$\begin{array}{l} \int_{0}^{1}\int_{0}^{1}...\int_{0}^{1}f\left(X_{1},X_{2},...,X_{n}\right)dX_{1}dX_{2}...dX_{n} \end{array}$$

to a one-dimensional integral.

Consider a multi-dimensional Fourier transformation of the function *f* that allows a variance-based decomposition of the samples in the input space along a curve defined as,


(7) $$\begin{array}{l} x_{i}\left(s\right)=G_{i}\left(sin\left(\omega_{i}s\right)\right);\text{for }i=1,2,....,n \end{array}$$

Where *x* = (*x*_1_, *x*_2_, …, *x*_*n*_) denotes a general point in the input space that has been sampled, ω_*i*_ is the *i*^*th*^ user-specified angular frequency corresponding to each input, *s*ϵℝ is a variable over the range (−∞, ∞), and *G*_*i*_ is the *i*^*th*^ transformation function (63). Varying *s* allows a multi-dimensional exploration of the input space since *x*_*is*_ are being simultaneously varied. Typically, we require *n* to be between 1,000 to 10,000 samples from the input space.

After applying the function *f*, the resulting scalar output (denoted by *Y*) produce different periodic functions based on different ω_*i*_. If the output *Y* is sensitive to changes in the *i*^*th*^ input factor, the periodic function of *Y* corresponding to frequency ω_*i*_ will have a high amplitude.

Specifically, we express the model, *Y* = *f*(*s*) = *f*(*x*_1_(*s*), *x*_2_(*s*), …, *x*_*n*_(*s*)) as a Fourier series;


(8) $$\begin{array}{l} Y=f\left(s\right)=\overset{\infty }{\underset{p=-\infty }{\sum }}A_{p}cos\left(ps\right)+B_{p}sin\left(ps\right) \end{array}$$

Using a domain of frequencies given by *p*ϵℤ = −∞, …, −1, 0, 1, …, ∞, then the Fourier coefficients *A*_*p*_ and *B*_*p*_ are defined as,


(9) $$\begin{array}{l} A_{p}=\frac{1}{2\pi }\int_{-\pi }^{\pi }f\left(s\right)cos\left(ps\right)ds \end{array}$$

and


(10) $$\begin{array}{l} B_{p}=\frac{1}{2\pi }\int_{-\pi }^{\pi }f\left(s\right)sin\left(ps\right)ds \end{array}$$

Therefore, the mean and variance of *Y* in Equation (8) can be approximated, respectively, as,


(11) $$\begin{array}{l} E\left(Y\right)\approx \frac{1}{2\pi }\int_{-\pi }^{\pi }f\left(s\right)ds \end{array}$$

and


(12) $$\begin{array}{l} Var\left(Y\right)\approx \frac{1}{2\pi }\int_{-\pi }^{\pi }f^{2}\left(s\right)ds-E^{2}\left(Y\right) \end{array}$$

Further, by applying the Parseval's theorem (64) to the approximations of the mean Equation (11) and the variance Equation (12), we can get,


(13) $$\begin{array}{l} Var\left(Y\right)\approx 2\overset{\infty }{\underset{p=1}{\sum }}\left(A_{p}^{2}+B_{p}^{2}\right) \end{array}$$

Thus, the FAST first-order sensitivity index (*SI*_*i*_) can be defined as,


(14) $$\begin{array}{l} SI_{i}=\frac{Var\left(Y_{i}\right)}{Var\left(Y\right)}\\ \;=\frac{2\sum_{q=1}^{\infty }\left(A_{q.\omega_{i}}^{2}+B_{q.\omega_{i}}^{2}\right)}{2\sum_{p=1}^{\infty }\left(A_{p}^{2}+B_{p}^{2}\right)}\\ \;\approx \frac{\sum_{q=1}^{M}\left(A_{q.\omega_{i}}^{2}+B_{q.\omega_{i}}^{2}\right)}{\sum_{i=1}^{n}\sum_{q=1}^{M}\left(A_{q.\omega_{i}}^{2}+B_{q.\omega_{i}}^{2}\right)} \end{array}$$

where *M* denotes the maximum harmonic (usually about 4 or 6) (65). The first-order sensitivity index *SI*_*i*_ represent the fraction of the model output variance due to the input variable (*X*_*i*_). A large index (i.e., *SI*_*i*_ > 0.1) means a significant first-order effect (61).

#### 2.4.2. Computation of the Total-Order Sensitivity Index (*SI*_*T*_*i*__)

Theoretically, the eFAST method (a GSA method) can compute sensitivity index of any order, as was given by Saltelli and colleagues (15). However, the computation for high order effects is cumbersome when the number of input parameters is large. A simple way for computation of the total order effect of each input parameter was therefore proposed by Homma and Saltelli (66), and it is summarized below Equation (15). Accordingly, the total-order sensitivity index (*SI*_*T*_*i*__) can be defined as the summed sensitivity index of the entire complementary set of parameters (i.e., all parameters except *i*) using their identification frequencies. Therefore, *SI*_*T*_*i*__ is then calculated as the remaining variance after the contribution of the complementary set (*s*_*c*_*i*__) is removed.


(15) $$\begin{array}{l} SI_{T_{i}}=SI_{i}-SI_{i,c_{i}}\\ \;=SI_{i}\left(1-SI_{c_{i}}\right)\\ \;=1-SI_{c_{i}} \end{array}$$

Where the *SI*_*i*_ and *SIi, c*_*i*_ represents the first-order and high-order effects respectively. *SI*_*c*_*i*__ is the sum of all the *SI*_*i*_1_, *i*_2_, …, *i*_*s*__ terms that excludes the index (*i*). Therefore, the *SI*_*T*_*i*__ includes the higher-order, nonlinear interactions between the parameter of interest and complementary set of parameters. A large index (i.e., *SI*_*T*_*i*__ > 0.1) means a significant total-order effect (61).

## 3. Results

### 3.1. Ascaris lumbricoides

The calculated sensitivity index for each parameter for the case of *Ascaris lumbriocoides* is presented in Table 3. Both the first-order (*SI*_*i*_) and total-order (*SI*_*T*_*i*__) sensitivity indices for each host is presented. From the table, WASH coverage (ϕ) was the most influential parameter across all the hosts, while population of the PSAC (*n*_*p*_), fecundity parameter for PSAC (λ_*p*_), and proportion of PSAC treated (*g*_*p*_) were the least influential parameters among PSAC, SAC, adults and the infectious materials in the environment, respectively.

**Table 3:** The first and total-order sensitivity indices (SI) of each parameter calculated using eFAST method for the case of *Ascaris lumbricoides*.

	**PSAC (Mp)**		**SAC (Mc)**		**Adults (Ma)**		**Infectious materials (L)**	
**Parameters**	***SI*_*i*_**	***SI*_*T*_*i*__**	***SI*_*i*_**	***SI*_*T*_*i*__**	***SI*_*i*_**	***SI*_*T*_*i*__**	***SI*_*i*_**	***SI*_*T*_*i*__**
β_*p*_	0.1701	0.1755	0.0989	0.0994	0.0669	0.0673	0.1263	0.1271
β_*c*_	0.0977	0.0981	0.1290	0.1367	0.0787	0.0791	0.1047	0.1050
β_*a*_	0.0647	0.0652	0.0649	0.0657	0.1202	0.1322	0.0893	0.0904
μ	0.4005	0.4526	0.3637	0.4182	0.4255	0.4918	0.3453	0.3884
μ_*L*_	0.1210	0.1286	0.0859	0.0925	0.0748	0.0802	0.1235	0.1334
*n*_*p*_	0.0364	0.0367	0.0275	0.0278	0.0320	0.0323	0.0496	0.0501
*n*_*c*_	0.0453	0.0463	0.0671	0.0689	0.0324	0.0328	0.0700	0.0715
*n*_*a*_	0.1031	0.1067	0.1091	0.1127	0.1088	0.1139	0.1358	0.1405
λ_*p*_	0.0572	0.0577	0.0312	0.0316	0.0252	0.0255	0.0680	0.0686
λ_*c*_	0.0881	0.0940	0.1497	0.1594	0.0675	0.0731	0.1336	0.1434
λ_*a*_	0.2434	0.2649	0.2465	0.2725	0.2588	0.2853	0.2970	0.3299
*k*	0.0769	0.0785	0.0640	0.0661	0.0669	0.0688	0.0922	0.0940
γ	0.1759	0.1855	0.1718	0.1823	0.1716	0.1827	0.1819	0.1939
ϕ	0.5603	0.6762	0.5680	0.6913	0.5409	0.6897	0.5458	0.6956
τ	0.1431	0.1538	0.1772	0.1889	0.1500	0.1621	0.1636	0.1740
*g*_*p*_	0.0669	0.0688	0.0308	0.0310	0.0286	0.0288	0.0405	0.0407
*g*_*c*_	0.0389	0.0399	0.0520	0.0556	0.0329	0.0338	0.0705	0.0727
*g*_*a*_	0.1440	0.1528	0.1606	0.1704	0.2301	0.2476	0.1889	0.2019
*h*	0.2694	0.3013	0.2697	0.2998	0.2848	0.3201	0.2696	0.2979

*We assumed a sampling size of n = 1, 000 and time t = 100 years. The SI presented here were the average values for the time interval*.

**Figure 1:**
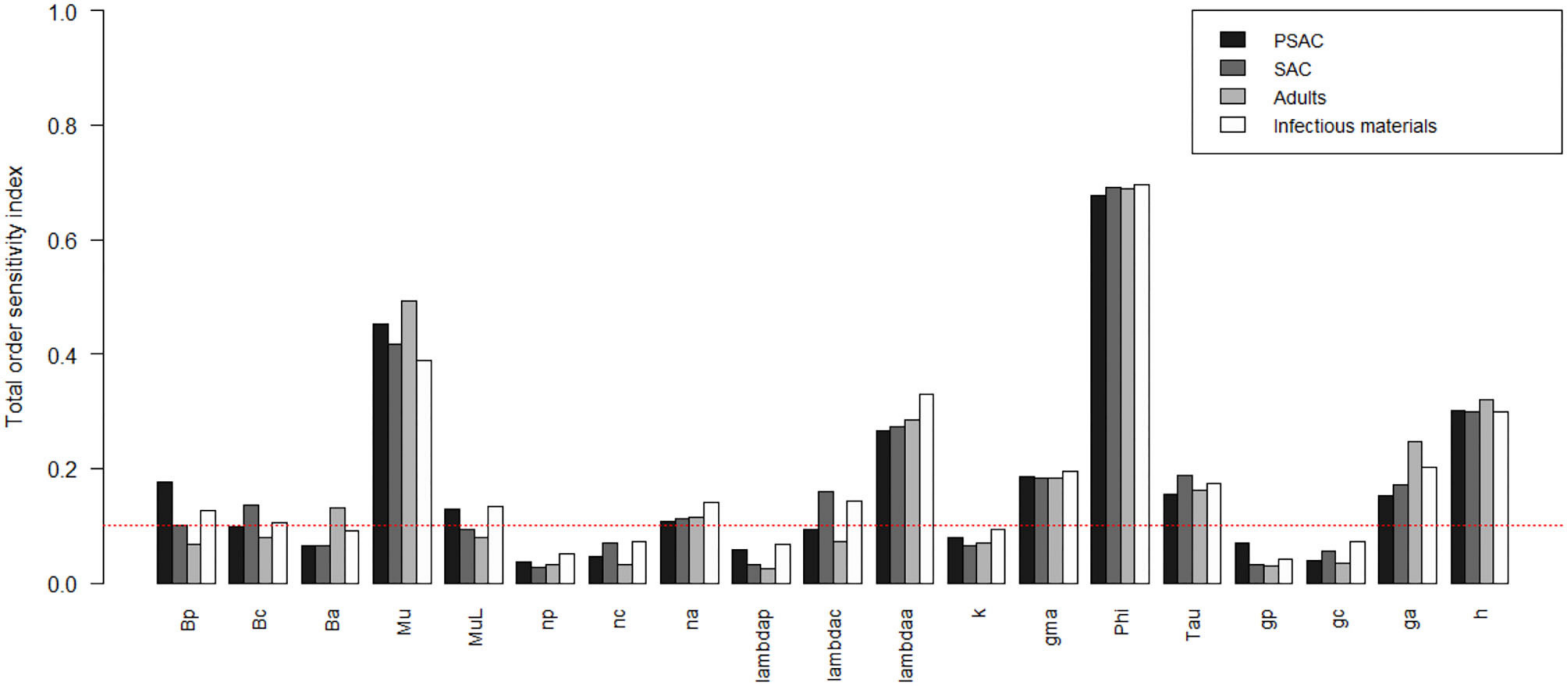
Comparison of the total-order sensitivity index of the parameters among the hosts using the eFAST method of sensitivity analysis for the case of *Ascaris lumbricoides*. The greater the sensitivity index, the more critical the parameter is to the model. The red dotted line indicated the cut-off sensitivity index value (SI = 0.1) above which a parameter was deemed significantly influential to the model outcome. *Bp*: PSAC infection transmission rate, *Bc*: SAC infection transmission rate, *Ba*: adults infection transmission rate, *Mu*: mature worm mortality rate, *MuL*: infectious materials mortality rate, *np*: PSAC population proportion, *nc*: SAC population proportion, *na*: adults population proportion, *lambdap*: relative contributions by PSAC, *lambdac*: relative contributions by SAC, *lambdaa*: relative contributions by adults, *k*: over-dispersion parameter, *gma*: strength of density dependence of worm egg production, *phi*: WASH effect, *Tau*: interval between treatment rounds per year, *gp*: proportion of PSAC treated, *gc*: proportion of SAC treated, *ga*: proportion of adults treated, and *h*: drug efficacy.

The comparison of the total-order sensitivity index (*SI*_*T*_*i*__) for each parameter among the hosts is outlined in Figure 1. From the figure, ϕ was the most influential (sensitive) parameter among all the hosts, followed by μ, *h*, λ_*a*_, *g*_*a*_, γ, τ, and then *n*_*a*_. Other parameters like β_*p*_, β_*c*_, β_*a*_, μ_*L*_, and λ_*c*_ were only influential in some specific hosts, directly related to these parameters, but not in all the hosts. However, parameters like *n*_*p*_, *n*_*c*_, λ_*p*_, *k*, *g*_*p*_, and *g*_*c*_ were not influential, or rather did not meet the pre-defined cut-off value of SI = 0.1, and hence did not contribute significantly to the model outcome (i.e., elimination of the worm burden). Additionally, the Supplementary Figure S1 shows the most influential (sensitive) parameters as indicated by both first and total order indices and compared among all the hosts and infectious materials.

**Figure 2:**
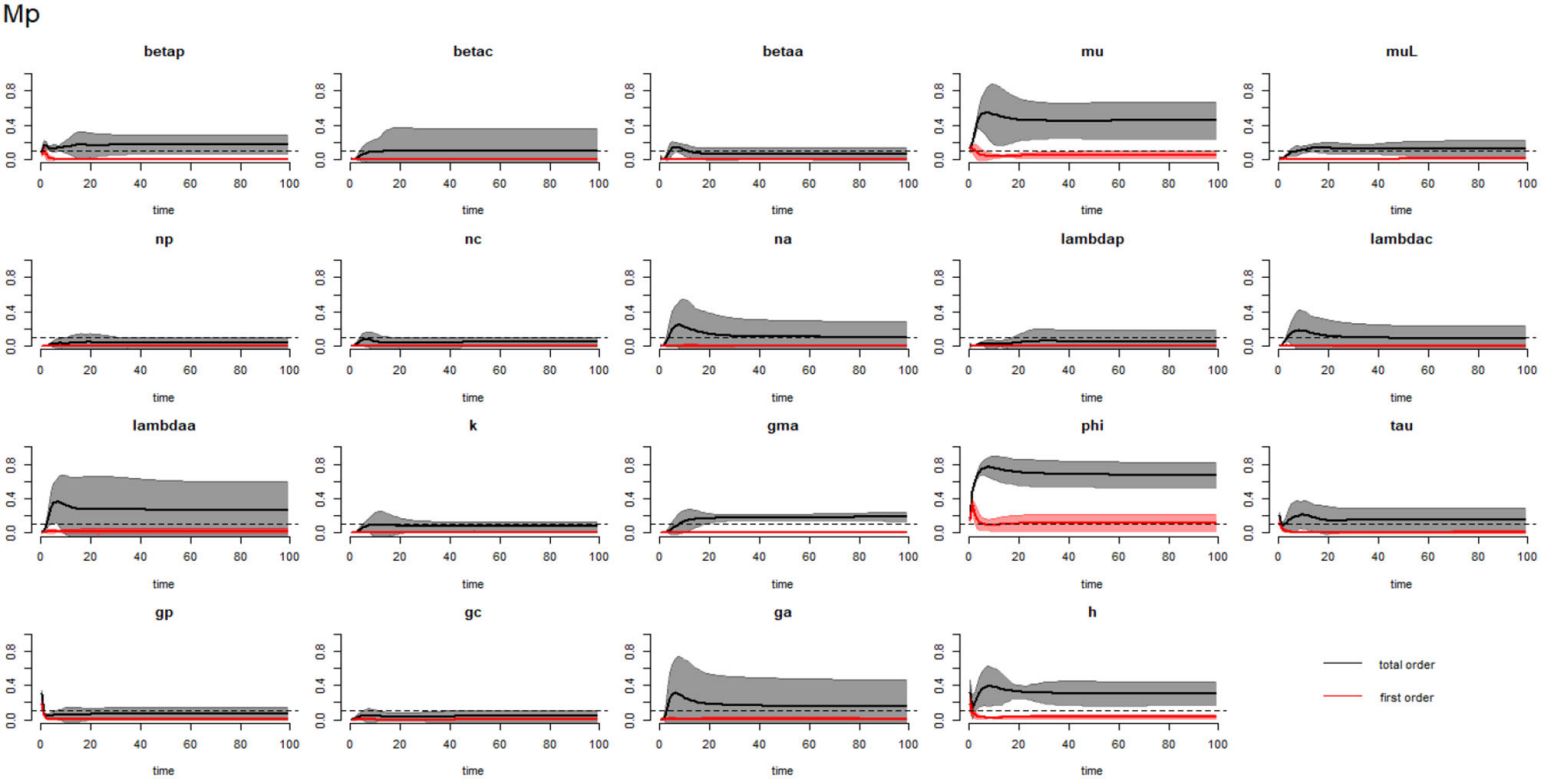
Plot of the first (solid red line with cloudy areas showing confidence intervals) and total-order (solid black line with cloudy areas showing confidence intervals) sensitivity indices of the parameters of PSAC (Mp) for the case of *Ascaris lumbricoides*. *betap*: PSAC infection transmission rate, *betac*: SAC infection transmission rate, *betaa*: adults infection transmission rate, *mu*: mature worm mortality rate, *muL*: infectious materials mortality rate, *np*: PSAC population proportion, *nc*: SAC population proportion, *na*: adults population proportion, *lambdap*: relative contributions by PSAC, *lambdac*: relative contributions by SAC, *lambdaa*: relative contributions by adults, *k*: over-dispersion parameter, *gma*: strength of density dependence of worm egg production, *phi*: WASH effect, *tau*: interval between treatment rounds per year, *g*_*p*_: proportion of PSAC treated, *g*_*c*_: proportion of SAC treated, *g*_*a*_: proportion of adults treated, and *h*: drug efficacy. The black dotted line indicated the cut-off sensitivity index value (SI = 0.1) above which a parameter was deemed significantly influential to the model outcome.

**Figure 3:**
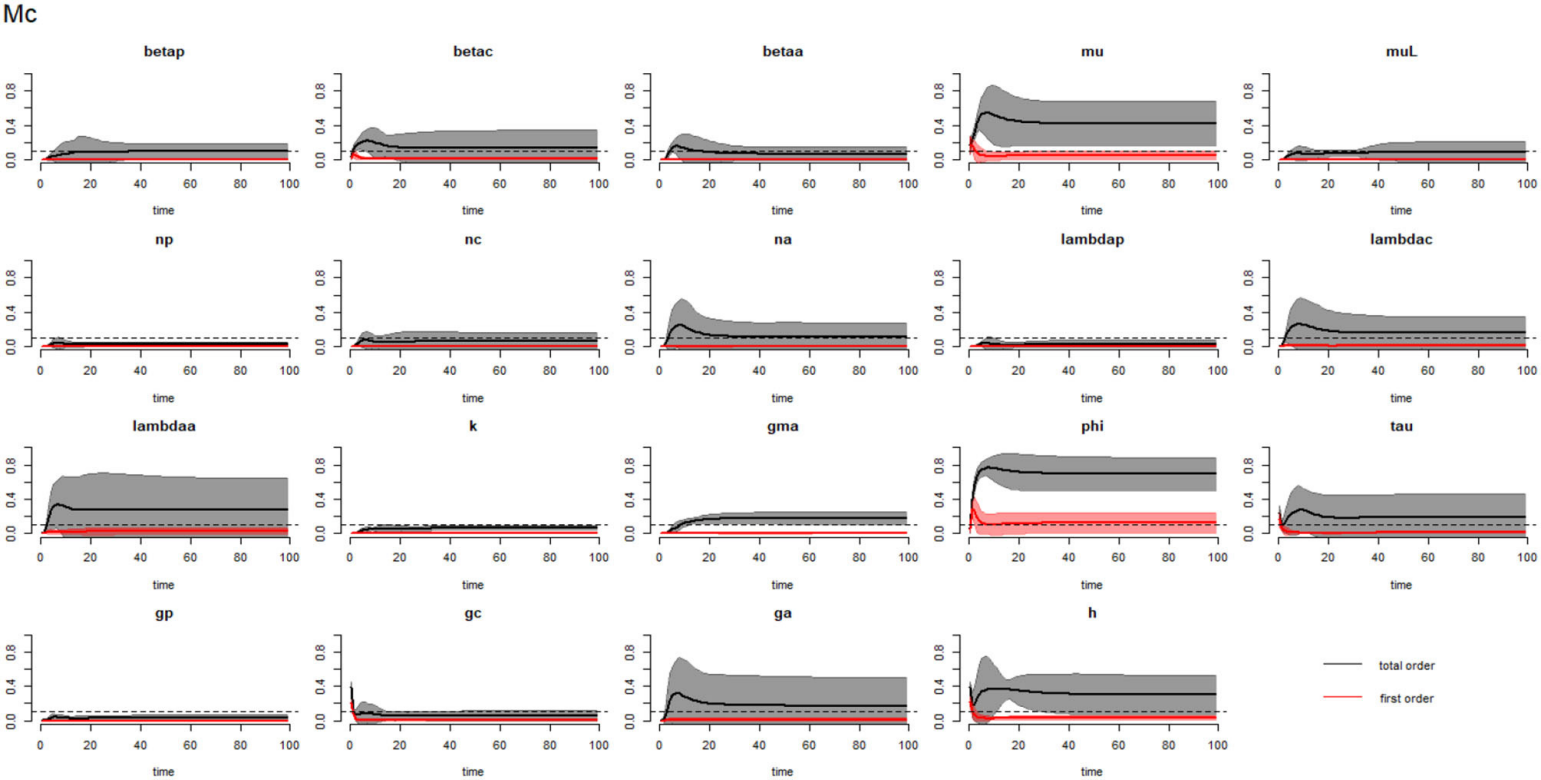
Plot of the first (solid red line with cloudy areas showing confidence intervals) and total-order (solid black line with cloudy areas showing confidence intervals) sensitivity indices of the parameters of SAC (Mc) for the case of *Ascaris lumbricoides*. *betap*: PSAC infection transmission rate, *betac*: SAC infection transmission rate, *betaa*: adults infection transmission rate, *mu*: mature worm mortality rate, *muL*: infectious materials mortality rate, *np*: PSAC population proportion, *nc*: SAC population proportion, *na*: adults population proportion, *lambdap*: relative contributions by PSAC, *lambdac*: relative contributions by SAC, *lambdaa*: relative contributions by adults, *k*: over-dispersion parameter, *gma*: strength of density dependence of worm egg production, *phi*: WASH effect, *tau*: interval between treatment rounds per year, *g*_*p*_: proportion of PSAC treated, *g*_*c*_: proportion of SAC treated, *g*_*a*_: proportion of adults treated, and *h*: drug efficacy. The black dotted line indicated the cut-off sensitivity index value (SI = 0.1) above which a parameter was deemed significantly influential to the model outcome.

**Figure 4:**
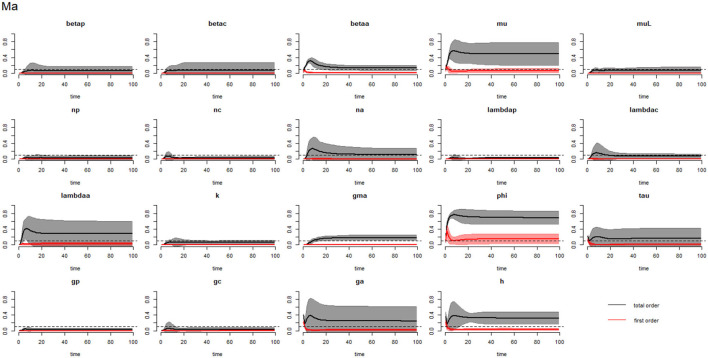
Plot of the first (solid red line with cloudy areas showing confidence intervals) and total-order (solid black line with cloudy areas showing confidence intervals) sensitivity indices of the parameters of adults (Ma) for the case of *Ascaris lumbricoides*. *betap*: PSAC infection transmission rate, *betac*: SAC infection transmission rate, *betaa*: adults infection transmission rate, *mu*: mature worm mortality rate, *muL*: infectious materials mortality rate, *np*: PSAC population proportion, *nc*: SAC population proportion, *na*: adults population proportion, *lambdap*: relative contributions by PSAC, *lambdac*: relative contributions by SAC, *lambdaa*: relative contributions by adults, *k*: over-dispersion parameter, *gma*: strength of density dependence of worm egg production, *phi*: WASH effect, *tau*: interval between treatment rounds per year, *g*_*p*_: proportion of PSAC treated, *g*_*c*_: proportion of SAC treated, *g*_*a*_: proportion of adults treated, and *h*: drug efficacy. The black dotted line indicated the cut-off sensitivity index value (SI = 0.1) above which a parameter was deemed significantly influential to the model outcome.

Figures 2–4 compare the first-order (*SI*_*i*_) and total-order (*SI*_*T*_*i*__) sensitivity indices for each parameter for PSAC, SAC, and adults respectively, while that for infectious materials is given in the supplementary file (Supplementary Figure S2). All parameters, except ϕ, were not influential (or rather did not meet the SI = 0.1 cut-off value) if we only consider the first-order sensitivity index (i.e., the influence of a single parameter on its own). However, several parameters showed significant influence (sensitivity) when we considered them in the presence of others (total-order sensitivity) (Figure 1).

### 3.2. Hookworm

The calculated sensitivity index for each parameter for the case of hookworm is presented in Table 4. Both the first-order (*SI*_*i*_) and total-order (*SI*_*T*_*i*__) sensitivity indices for each host is presented. From the table, the adult parasite death rate (μ) was the most influential parameter among the PSAC, adults and infectious materials in the environment, while WASH coverage (ϕ) was the most influential parameter among the SAC. The population of the PSAC (*n*_*p*_) was the least influential parameter across all the hosts.

**Table 4:** The first and total-order sensitivity indices (SI) of each parameter calculated using eFAST method for the case of hookworm.

	**PSAC (Mp)**		**SAC (Mc)**		**Adults (Ma)**		**Infectious materials (L)**	
**Parameters**	***SI*_*i*_**	***SI*_*T*_*i*__**	***SI*_*i*_**	***SI*_*T*_*i*__**	***SI*_*i*_**	***SI*_*T*_*i*__**	***SI*_*i*_**	***SI*_*T*_*i*__**
β_*p*_	0.2366	0.2429	0.1877	0.1890	0.1516	0.1527	0.2023	0.2038
β_*c*_	0.0848	0.0861	0.1112	0.1162	0.0793	0.0803	0.0974	0.0993
β_*a*_	0.0877	0.0892	0.0649	0.0657	0.0773	0.0843	0.1005	0.1054
μ	0.5748	0.6580	0.5575	0.6448	0.5664	0.6661	0.5524	0.6298
μ_*L*_	0.0665	0.0670	0.0788	0.0795	0.0311	0.0313	0.0577	0.0582
*n*_*p*_	0.0159	0.0161	0.0245	0.0246	0.0190	0.0191	0.0358	0.0360
*n*_*c*_	0.0203	0.0208	0.0281	0.0287	0.0207	0.0212	0.0392	0.0399
*n*_*a*_	0.0754	0.0774	0.0763	0.0782	0.0685	0.0704	0.0896	0.0930
λ_*p*_	0.0515	0.0518	0.0278	0.0279	0.0213	0.0214	0.0525	0.0527
λ_*c*_	0.0810	0.0838	0.1675	0.1734	0.0538	0.0561	0.1134	0.1184
λ_*a*_	0.3169	0.3440	0.3299	0.3637	0.3387	0.3731	0.3804	0.4231
*k*	0.0569	0.0576	0.0675	0.0685	0.0535	0.0545	0.0745	0.0755
γ	0.1685	0.1757	0.1677	0.1751	0.1677	0.1765	0.1814	0.1904
ϕ	0.5695	0.6435	0.5790	0.6459	0.5590	0.6593	0.5495	0.6293
τ	0.1245	0.1314	0.1798	0.1883	0.1151	0.1218	0.1351	0.1403
*g*_*p*_	0.0586	0.0612	0.0390	0.0394	0.0308	0.0310	0.0380	0.0384
*g*_*c*_	0.0453	0.0461	0.0392	0.0420	0.0282	0.0288	0.0585	0.0598
*g*_*a*_	0.1290	0.1363	0.1450	0.1530	0.1611	0.1717	0.1317	0.1394
*h*	0.2629	0.2835	0.3180	0.3415	0.2446	0.2680	0.2472	0.2660

*We assumed a sampling size of n = 1000 and time t = 100years. The SI presented here were the average values for the time interval*.

**Figure 5:**
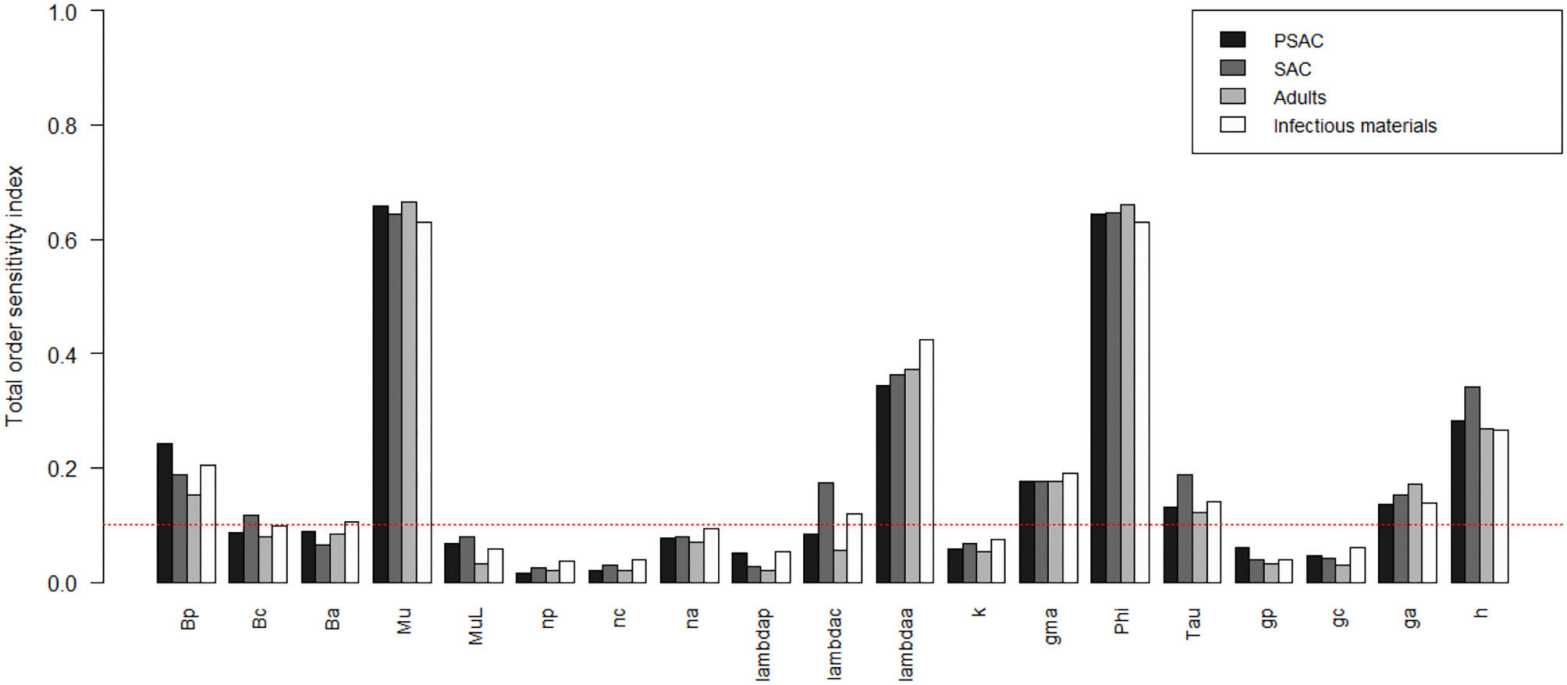
Comparison of the total-order sensitivity index of the parameters among the hosts using the eFAST method of sensitivity analysis for the case of hookworm. The greater the sensitivity index, the more critical the parameter is to the model. The red dotted line indicated the cut-off sensitivity index value (SI = 0.1) above which a parameter was deemed significantly influential to the model outcome. *Bp*: PSAC infection transmission rate, *Bc*: SAC infection transmission rate, *Ba*: adults infection transmission rate, *Mu*: mature worm mortality rate, *MuL*: infectious materials mortality rate, *np*: PSAC population proportion, *nc*: SAC population proportion, *na*: adults population proportion, *lambdap*: relative contributions by PSAC, *lambdac*: relative contributions by SAC, *lambdaa*: relative contributions by adults, *k*: over-dispersion parameter, *gma*: strength of density dependence of worm egg production, *phi*: WASH effect, *Tau*: interval between treatment rounds per year, *gp*: proportion of PSAC treated, *gc*: proportion of SAC treated, *ga*: proportion of adults treated, and *h*: drug efficacy.

The comparison of the total-order sensitivity index (*SI*_*T*_*i*__) for each parameter by the hosts is outlined in Figure 5. From the figure, μ was the most influential (sensitive) parameter among all the hosts, followed by ϕ, λ_*a*_, *h*, β_*p*_, γ, *g*_*a*_, and then τ. Other parameters like β_*c*_, β_*a*_, and λ_*c*_ were only influential in some specific hosts, directly related to these parameters, but not in all the hosts. However, parameters like *n*_*p*_, *n*_*c*_, *n*_*a*_, μ_*L*_, λ_*p*_, *k*, *g*_*p*_, and *g*_*c*_ were not influential, or rather did not meet the pre-defined cut-off value of SI = 0.1, and hence did not contribute significantly to the model outcome (i.e., elimination of the worm burden). Additionally, the Supplementary Figure S3 shows the most influential (sensitive) parameters as indicated by both first and total order indices and compared among all the hosts and infectious materials.

**Figure 6:**
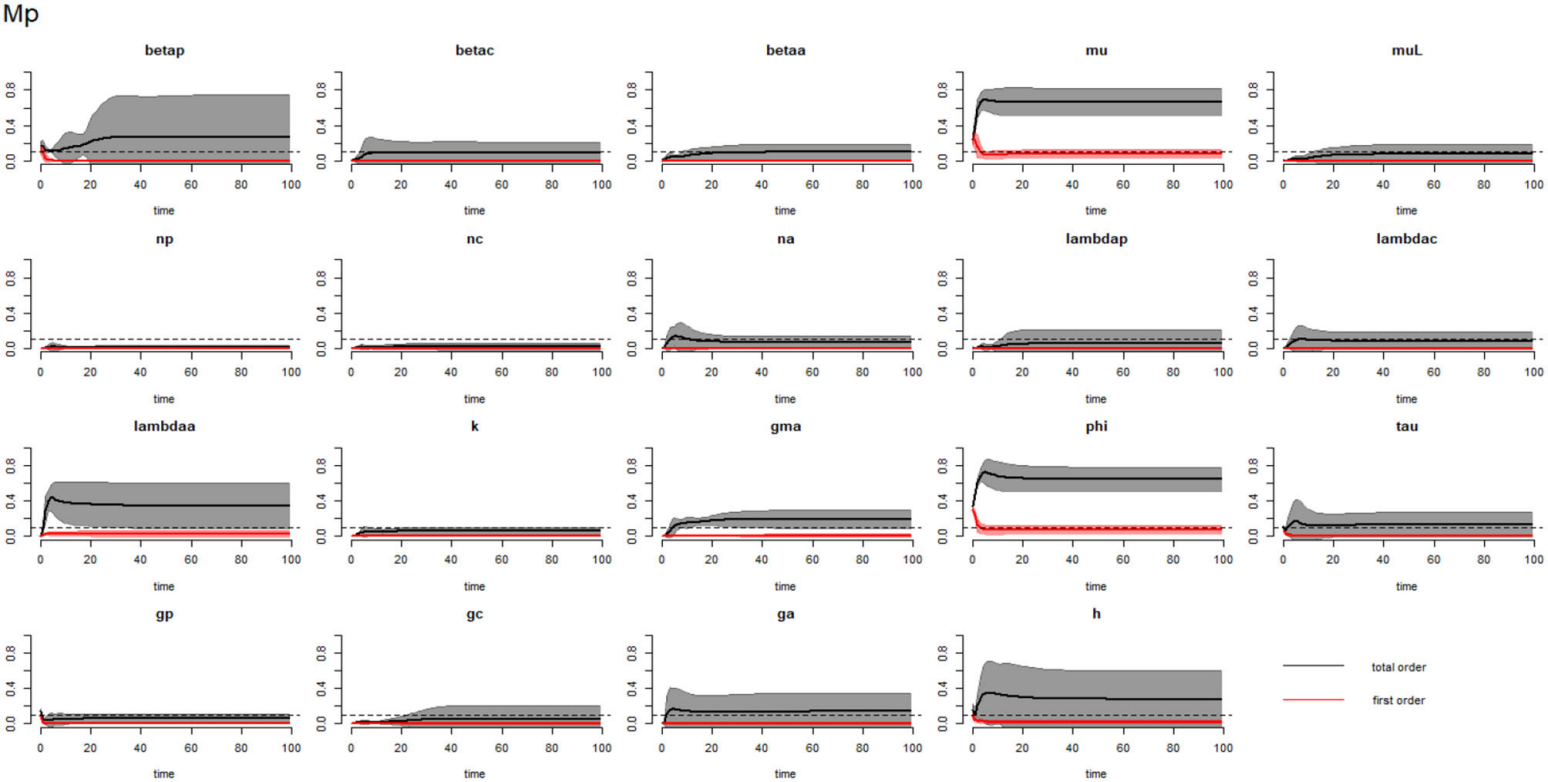
Plot of the first (solid red line with cloudy areas showing confidence intervals) and total-order (solid black line with cloudy areas showing confidence intervals) sensitivity indices of the parameters of PSAC (Mp) for the case of hookworm. *betap*: PSAC infection transmission rate, *betac*: SAC infection transmission rate, *betaa*: adults infection transmission rate, *mu*: mature worm mortality rate, *muL*: infectious materials mortality rate, *np*: PSAC population proportion, *nc*: SAC population proportion, *na*: adults population proportion, *lambdap*: relative contributions by PSAC, *lambdac*: relative contributions by SAC, *lambdaa*: relative contributions by adults, *k*: over-dispersion parameter, *gma*: strength of density dependence of worm egg production, *phi*: WASH effect, *tau*: interval between treatment rounds per year, *g*_*p*_: proportion of PSAC treated, *g*_*c*_: proportion of SAC treated, *g*_*a*_: proportion of adults treated, and *h*: drug efficacy. The black dotted line indicated the cut-off sensitivity index value (SI = 0.1) above which a parameter was deemed significantly influential to the model outcome.

**Figure 7:**
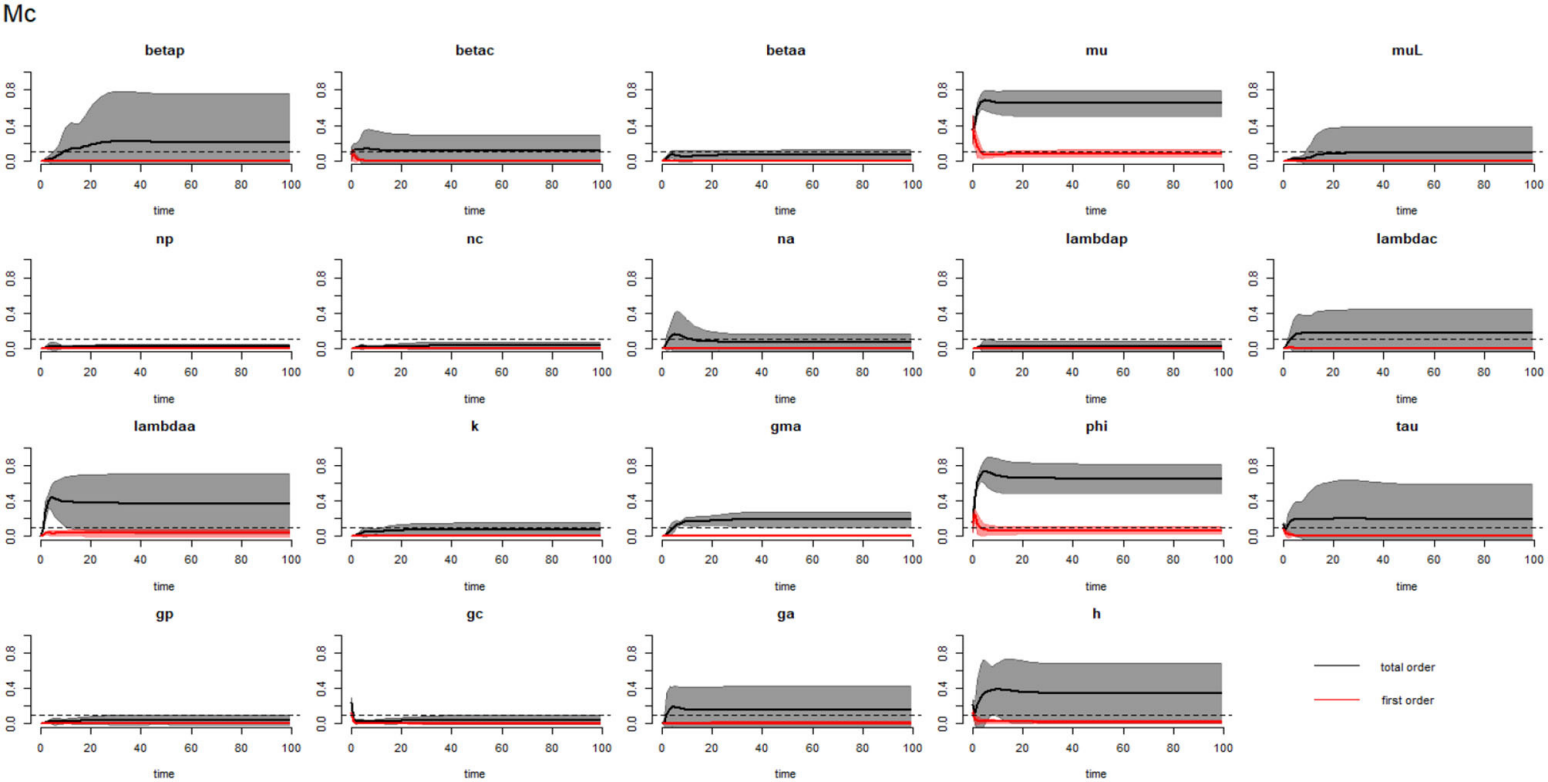
Plot of the first (solid red line with cloudy areas showing confidence intervals) and total-order (solid black line with cloudy areas showing confidence intervals) sensitivity indices of the parameters of SAC (Mc) for the case of hookworm. *betap*: PSAC infection transmission rate, *betac*: SAC infection transmission rate, *betaa*: adults infection transmission rate, *mu*: mature worm mortality rate, *muL*: infectious materials mortality rate, *np*: PSAC population proportion, *nc*: SAC population proportion, *na*: adults population proportion, *lambdap*: relative contributions by PSAC, *lambdac*: relative contributions by SAC, *lambdaa*: relative contributions by adults, *k*: over-dispersion parameter, *gma*: strength of density dependence of worm egg production, *phi*: WASH effect, *tau*: interval between treatment rounds per year, *g*_*p*_: proportion of PSAC treated, *g*_*c*_: proportion of SAC treated, *g*_*a*_: proportion of adults treated, and *h*: drug efficacy. The black dotted line indicated the cut-off sensitivity index value (SI = 0.1) above which a parameter was deemed significantly influential to the model outcome.

**Figure 8:**
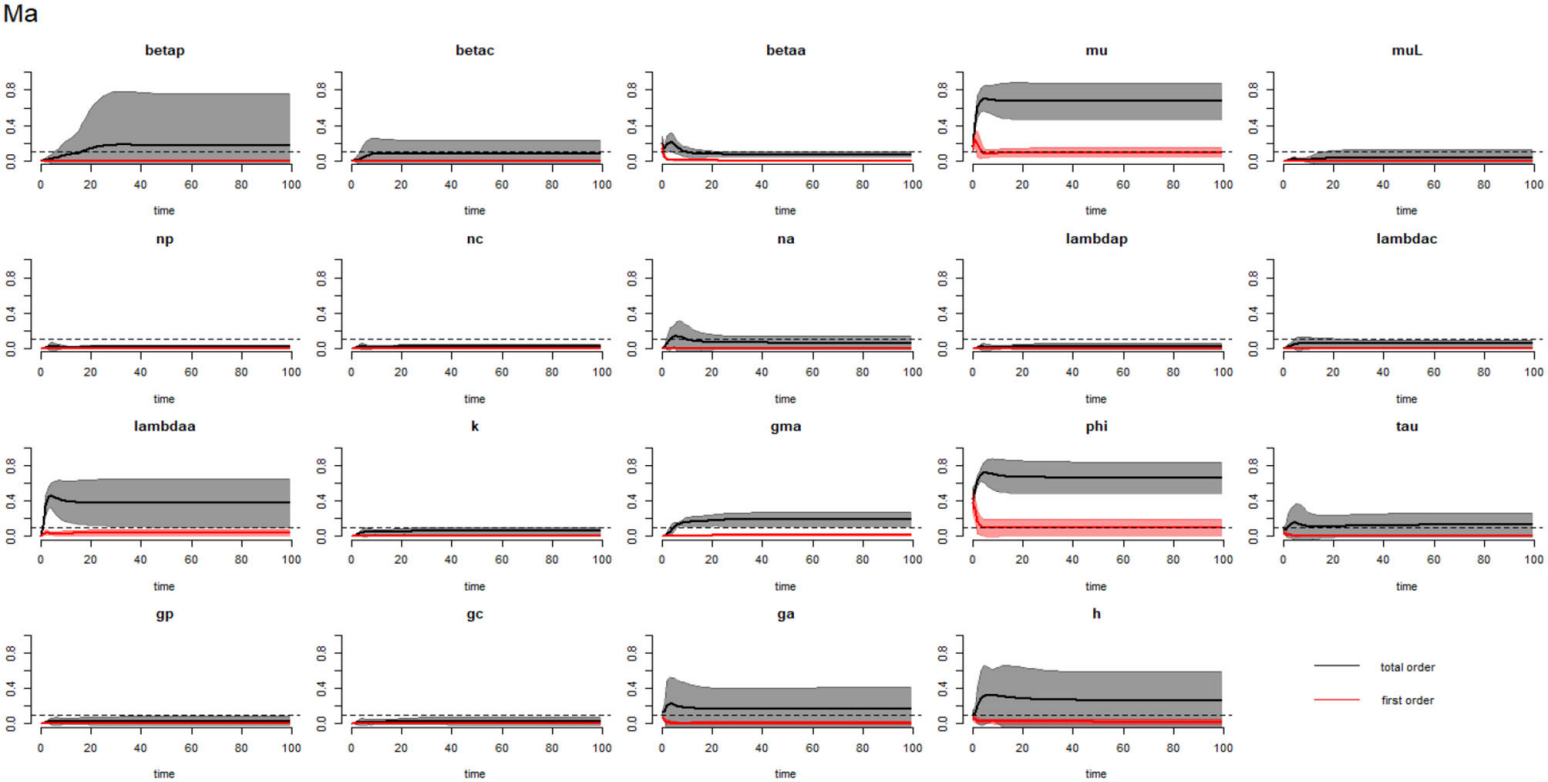
Plot of the first (solid red line with cloudy areas showing confidence intervals) and total-order (solid black line with cloudy areas showing confidence intervals) sensitivity indices of the parameters of adults (Ma) for the case of hookworm. *betap*: PSAC infection transmission rate, *betac*: SAC infection transmission rate, *betaa*: adults infection transmission rate, *mu*: mature worm mortality rate, *muL*: infectious materials mortality rate, *np*: PSAC population proportion, *nc*: SAC population proportion, *na*: adults population proportion, *lambdap*: relative contributions by PSAC, *lambdac*: relative contributions by SAC, *lambdaa*: relative contributions by adults, *k*: over-dispersion parameter, *gma*: strength of density dependence of worm egg production, *phi*: WASH effect, *tau*: interval between treatment rounds per year, *g*_*p*_: proportion of PSAC treated, *g*_*c*_: proportion of SAC treated, *g*_*a*_: proportion of adults treated, and *h*: drug efficacy. The black dotted line indicated the cut-off sensitivity index value (SI = 0.1) above which a parameter was deemed significantly influential to the model outcome.

Figures 6–8 compare the first-order (*SI*_*i*_) and total-order (*SI*_*T*_*i*__) sensitivity indices for each parameter for PSAC, SAC, and adults respectively, while that for infectious materials is given in the supplementary file (Supplementary Figure S4). All parameters, except μ and ϕ, were not influential (or rather did not meet the SI = 0.1 cut-off value) if we only consider the first-order sensitivity index (i.e., the influence of a single parameter on its own). However, several parameters showed significant influence (sensitivity) when we considered them in the presence of others (total-order sensitivity) (Figure 5).

### 3.3. Trichuris trichiura

The calculated sensitivity index for each parameter for the case of *Trichuris trichiura* is presented in Table 5. Both the first-order (*SI*_*i*_) and total-order (*SI*_*T*_*i*__) sensitivity indices for each host is presented. From the table, WASH coverage (ϕ) was the most influential parameter across all the hosts. The least influential parameters were; proportion of SAC treated (*g*_*c*_), proportion of PSAC treated (*g*_*p*_) and fecundity parameter for PSAC (λ_*p*_), and these parameters had varied values across each of the hosts (i.e., PSAC, SAC, and adults) and the infectious materials.

**Table 5:** The first and total-order sensitivity indices (SI) of each parameter calculated using eFAST method for the case of *Trichuris trichiura*.

	**PSAC (Mp)**		**SAC (Mc)**		**Adults (Ma)**		**Infectious materials (L)**	
**Parameters**	***SI*_*i*_**	***SI*_*T*_*i*__**	***SI*_*i*_**	***SI*_*T*_*i*__**	***SI*_*i*_**	***SI*_*T*_*i*__**	***SI*_*i*_**	***SI*_*T*_*i*__**
β_*p*_	0.1331	0.1449	0.0471	0.0473	0.0507	0.0509	0.0524	0.0526
β_*c*_	0.0300	0.0302	0.0859	0.0949	0.0208	0.0209	0.0284	0.0288
β_*a*_	0.0428	0.0434	0.0569	0.0579	0.1269	0.1408	0.0622	0.0642
μ	0.3532	0.3903	0.3488	0.3911	0.3803	0.4261	0.2932	0.3274
μ_*L*_	0.0388	0.0405	0.0254	0.0266	0.0255	0.0267	0.0356	0.0388
*n*_*p*_	0.0405	0.0407	0.0304	0.0305	0.0240	0.0241	0.0344	0.0346
*n*_*c*_	0.0263	0.0266	0.0303	0.0308	0.0177	0.0178	0.0248	0.0258
*n*_*a*_	0.0992	0.1023	0.1103	0.1136	0.1214	0.1256	0.1270	0.1319
λ_*p*_	0.0313	0.0319	0.0196	0.0200	0.0146	0.0148	0.0222	0.0227
λ_*c*_	0.0935	0.0982	0.1536	0.1608	0.0648	0.0696	0.1086	0.1187
λ_*a*_	0.2658	0.2876	0.2555	0.2799	0.2953	0.3206	0.3047	0.3423
*k*	0.0939	0.0960	0.0766	0.0789	0.0810	0.0833	0.0840	0.0865
γ	0.1486	0.1548	0.1467	0.1527	0.1476	0.1549	0.1533	0.1614
ϕ	0.5558	0.6513	0.5592	0.6590	0.5386	0.6504	0.5281	0.6398
τ	0.1905	0.2112	0.2281	0.2512	0.1960	0.2199	0.1824	0.2007
*g*_*p*_	0.0697	0.0804	0.0187	0.0189	0.0237	0.0238	0.0250	0.0253
*g*_*c*_	0.0199	0.0206	0.0793	0.0927	0.0249	0.0259	0.0413	0.0439
*g*_*a*_	0.1892	0.2052	0.1979	0.2156	0.3180	0.3531	0.2632	0.2882
*h*	0.3944	0.4501	0.4122	0.4722	0.3744	0.4347	0.3535	0.4088

*We assumed a sampling size of n = 1000 and time t = 100years. The SI presented here were the average values for the time interval*.

**Figure 9:**
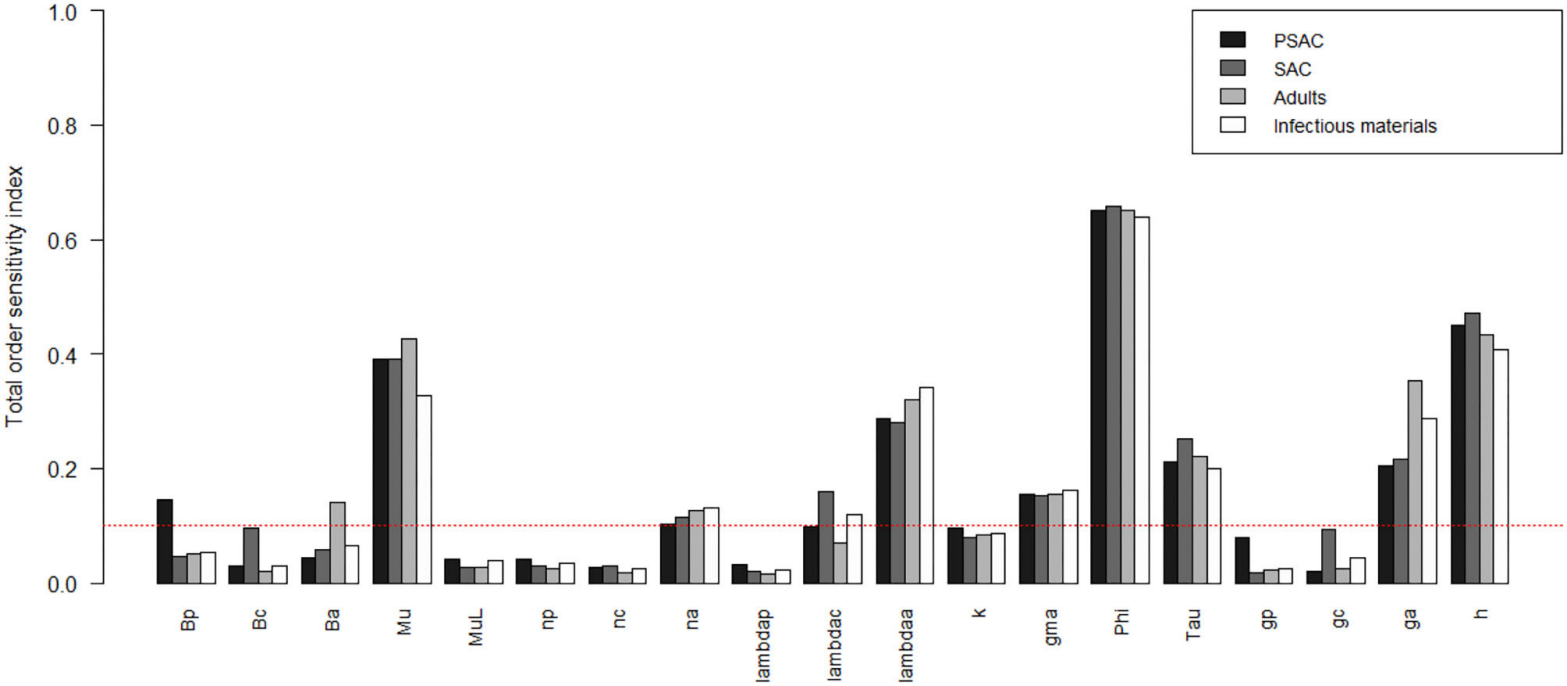
Comparison of the total-order sensitivity index of the parameters among the hosts using the eFAST method of sensitivity analysis for the case of *Trichuris trichiura*. The greater the sensitivity index, the more critical the parameters are for the model outcome. *Bp*: PSAC infection transmission rate, *Bc*: SAC infection transmission rate, *Ba*: adults infection transmission rate, *Mu*: mature worm mortality rate, *MuL*: infectious materials mortality rate, *np*: PSAC population proportion, *nc*: SAC population proportion, *na*: adults population proportion, *lambdap*: relative contributions by PSAC, *lambdac*: relative contributions by SAC, *lambdaa*: relative contributions by adults, *k*: over-dispersion parameter, *gma*: strength of density dependence of worm egg production, *phi*: WASH effect, *Tau*: interval between treatment rounds per year, *gp*: proportion of PSAC treated, *gc*: proportion of SAC treated, *ga*: proportion of adults treated, and *h*: drug efficacy.

The comparison of the total-order sensitivity index (*SI*_*T*_*i*__) for each parameter by the hosts is outlined in Figure 9. From the figure, ϕ was the most influential (sensitive) parameter among all the hosts, followed by *h*, μ, λ_*a*_, *g*_*a*_, τ, γ, and then *n*_*a*_. Other parameters like β_*p*_, β_*a*_, and λ_*c*_ were only influential in some specific hosts, directly related to these parameters, but not in all the hosts. However, parameters like β_*c*_, μ_*L*_, *n*_*p*_, *n*_*c*_, λ_*p*_, *k*, *g*_*p*_, and *g*_*c*_ were not influential, or rather did not meet the pre-defined cut-off value of SI = 0.1, and hence did not contribute significantly to the model outcome (i.e., elimination of the worm burden). Additionally, the Supplementary Figure S5 shows the most influential (sensitive) parameters as indicated by both first and total order indices and compared among all the hosts and infectious materials.

**Figure 10:**
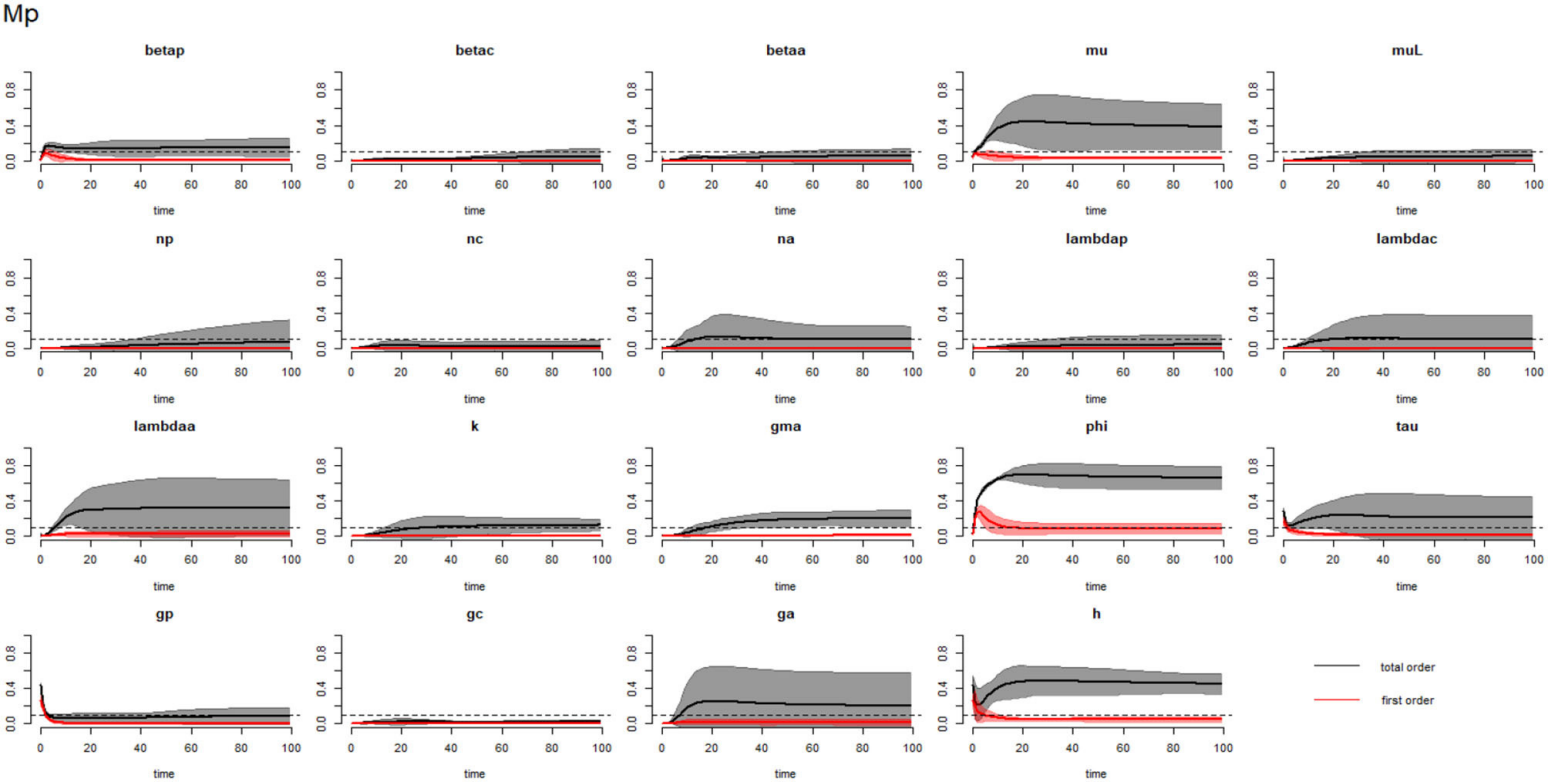
Plot of the first (solid red line with cloudy areas showing confidence intervals) and total-order (solid black line with cloudy areas showing confidence intervals) sensitivity indices of the parameters of PSAC (Mp) for the case of *Trichuris trichiura*. *betap*: PSAC infection transmission rate, *betac*: SAC infection transmission rate, *betaa*: adults infection transmission rate, *mu*: mature worm mortality rate, *muL*: infectious materials mortality rate, *np*: PSAC population proportion, *nc*: SAC population proportion, *na*: adults population proportion, *lambdap*: relative contributions by PSAC, *lambdac*: relative contributions by SAC, *lambdaa*: relative contributions by adults, *k*: over-dispersion parameter, *gma*: strength of density dependence of worm egg production, *phi*: WASH effect, *tau*: interval between treatment rounds per year, *g*_*p*_: proportion of PSAC treated, *g*_*c*_: proportion of SAC treated, *g*_*a*_: proportion of adults treated, and *h*: drug efficacy. The black dotted line indicated the cut-off sensitivity index value (SI = 0.1) above which a parameter was deemed significantly influential to the model outcome.

**Figure 11:**
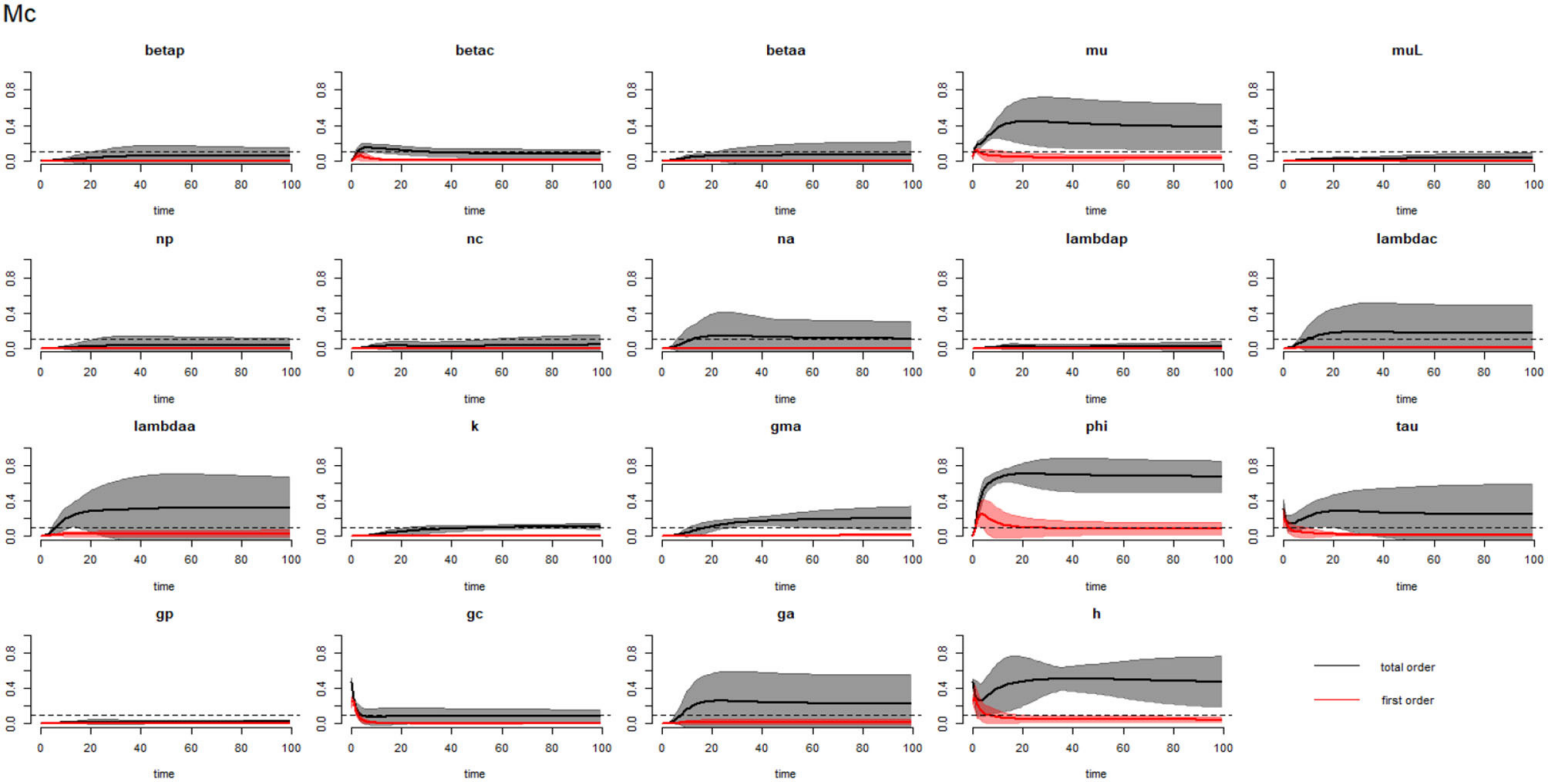
Plot of the first (solid red line with cloudy areas showing confidence intervals) and total-order (solid black line with cloudy areas showing confidence intervals) sensitivity indices of the parameters of SAC (Mc) for the case of *Trichuris trichiura*. *betap*: PSAC infection transmission rate, *betac*: SAC infection transmission rate, *betaa*: adults infection transmission rate, *mu*: mature worm mortality rate, *muL*: infectious materials mortality rate, *np*: PSAC population proportion, *nc*: SAC population proportion, *na*: adults population proportion, *lambdap*: relative contributions by PSAC, *lambdac*: relative contributions by SAC, *lambdaa*: relative contributions by adults, *k*: over-dispersion parameter, *gma*: strength of density dependence of worm egg production, *phi*: WASH effect, *tau*: interval between treatment rounds per year, *g*_*p*_: proportion of PSAC treated, *g*_*c*_: proportion of SAC treated, *g*_*a*_: proportion of adults treated, and *h*: drug efficacy. The black dotted line indicated the cut-off sensitivity index value (SI = 0.1) above which a parameter was deemed significantly influential to the model outcome.

**Figure 12:**
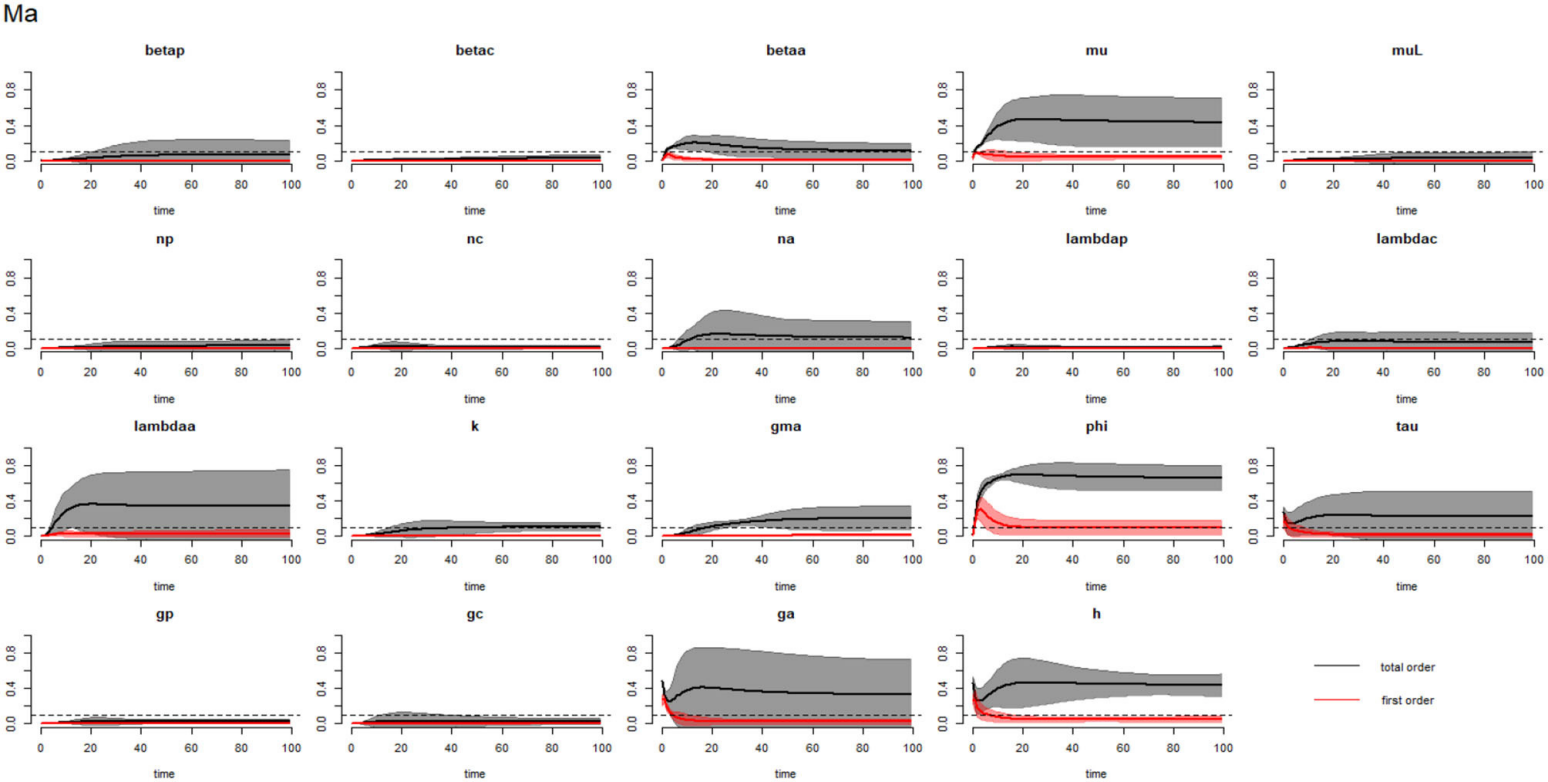
Plot of the first (solid red line with cloudy areas showing confidence intervals) and total-order (solid black line with cloudy areas showing confidence intervals) sensitivity indices of the parameters of adults (Ma) for the case of *Trichuris trichiura*. *betap*: PSAC infection transmission rate, *betac*: SAC infection transmission rate, *betaa*: adults infection transmission rate, *mu*: mature worm mortality rate, *muL*: infectious materials mortality rate, *np*: PSAC population proportion, *nc*: SAC population proportion, *na*: adults population proportion, *lambdap*: relative contributions by PSAC, *lambdac*: relative contributions by SAC, *lambdaa*: relative contributions by adults, *k*: over-dispersion parameter, *gma*: strength of density dependence of worm egg production, *phi*: WASH effect, *tau*: interval between treatment rounds per year, *g*_*p*_: proportion of PSAC treated, *g*_*c*_: proportion of SAC treated, *g*_*a*_: proportion of adults treated, and *h*: drug efficacy. The black dotted line indicated the cut-off sensitivity index value (SI = 0.1) above which a parameter was deemed significantly influential to the model outcome.

Figures 10–12 compare the first-order (*SI*_*i*_) and total-order (*SI*_*T*_*i*__) sensitivity indices for each parameter for PSAC, SAC and adults respectively, while that for infectious materials is given in supplementary file (Supplementary Figure S6). All parameters, except ϕ and *h*, were not influential (or rather did not meet the SI = 0.1 cut-off value) if we only consider the first-order sensitivity index (i.e., the influence of a single parameter on its own). However, several parameters showed significant influence (sensitivity) when we considered them in the presence of others (total-order sensitivity) (Figure 9).

## 4. Discussion

Recently, elimination of NTDs has gained focus and increased interest among the control programmes in the endemic countries around the globe (67–69). This interest has seen more fundings made available from international agencies for the donation of drugs, revamped (re)mapping of new transmission areas, consolidation of WASH efforts in the communities, and innovative ways of achieving global elimination (70, 71). Resources are becoming available for the treatment of STH infections through either school-based deworming (SBD) or community-based deworming (CBD) strategies (72). However, little mathematical modeling studies have been conducted to assess and investigate key parameters influencing transmission and elimination of STH infections. In this study, we assessed and estimated the sensitivities of key parameters influencing the elimination of STH infections in Kenya. The results of this study would be helpful in guiding the design and implementation of an efficient STH elimination strategy in the country, since it has clearly indicated which parameters are siginificantly influencing STH elimination and are thus worth investing in.

We performed a global sensitivity analysis of an STH-transmission model using 19 parameters thought to influence the transmission and elimination of STH infections in Kenya. These parameters can be conveniently broadly grouped as; (i) intervention-related, (ii) worm-related, (iii) population-related, and (iv) transmission and fecundity-related parameters. Intervention-related parameters analyzed included the combined effect of improved water source and sanitation (i.e., WASH coverage; ϕ), rounds of mass treatment (i.e., MDA) offered per year (τ), proportion of individuals treated in every treatment round (*g*_*i*_ for *i* = *p, c, a*), and the efficacy of the drug used during treatment (*h*). Worm-related parameters included mortality rate of the mature worms in the human host (μ), mortality rate of the free-living infectious materials in the environment (μ_*L*_), and the strength of the density dependence of worm egg production (γ) and the over-dispersion (aggregation) parameter of the worm burden distribution (*k*). Population-related parameters included the proportion of each host in the overall population (*n*_*i*_ for *i* = *p, c, a*). Transmission and fecundity-related parameters included infection transmission rate among each host (β_*i*_ for *i* = *p, c, a*) and the relative contribution (contamination) to the environment by each host (λ_*i*_ for *i* = *p, c, a*), respectively.

Sensitivity indices for each parameter were calculated and compared for each human host as well as for the infectious materials. SI values ranged from zero to one, with zero value indicating that the parameter had no influence (effect) on the model outcome (elimination), while the value one indicating that the parameter had a strong influence on the model outcome. First-order SI (*SI*_*i*_) values indicated the single influence of a particular parameter in the absence of the effect of the other parameters, while the total-order SI (*SI*_*T*_*i*__) values demonstrated the combined effect of a parameter taking into account the effect of other parameters. A cut-off value of SI = 0.1 was adopted (61), SI values above this cut-off were considered to be significantly influential to the model outcome with significance increasing with the increase in the SI value. On the other hand, SI values below the cut-off were considered to have non-significant (little) influence with zero value indicating no influence.

All the intervention-related parameters (ϕ, τ, and *h*) analyzed in this study were found to be significantly most influential to the model outcome for all the three parasites (*Ascaris lumbricoides*, hookworm and *Trichuris trichiura*). These results indicate that for a control programme to effectively eliminate these three major parasites, prioritization of WASH interventions and optimization of its coverage coupled with interventions that directly kill the adult worms in the human hosts (i.e., interventions like MDA to at-risk individuals) should always be optimized. This modeling results agree with past studies that have shown that the impact of an intervention strategy employed and its specific properties like the efficacy of the drug used and the number of times (rounds) the drug is administered, can be very sensitive to the reproduction number [*R*_*o*_: i.e., a summary parameter for the intensity of an infection transmission (73)] and the overall model outcome (33). If these parameters are optimized, then the impact of the intervention is enhanced implying faster elimination of the worm burden in the hosts (3). However, for the *g*_*i*_ for *i* = *p, c, a*, only *g*_*a*_ was significantly influential while *g*_*p*_ and *g*_*c*_ were not influential at all for any of the parasites. This could be explained by the fact that in this model, substantial proportion of the individuals considered were adults, implying that if greater proportion of adults were treated then they would certainly influence the elimination of the worms in the entire community (35).

Mixed impacts on sensitivity analysis regarding worm-related parameters were observed. μ and γ were the only single most important parameters across all the three parasites and hosts. Adult worm mortality rate highly influenced the model outcome since morbidity is related to the number of worms harbored, people with light intensity (few worms) usually do not suffer from the infection (74). Therefore, higher mortality rate indicate reduced worm burden in the host. On the other hand, μ_*L*_ was only sensitive among the PSAC as well as infectious materials in the environment and for the case of *Ascaris lumbricoides* only. This finding is supported by previous epidemiological studies that have shown high STH (especially *Ascaris lumbricoides*) burden among pre-school children (75). This high worm burden in these younger children is attributable to their frequent interaction with the contaminated environment especially when they are playing with the soil, eating soil (geophagy), or practicing open defecation (75, 76). Therefore, changes in the mortality rate of the infectious materials in the environment will certainly influence the level of worm burdens in the PSAC owing to their high interaction with the environment. However, from our results, *k* did not show any significant importance for any parasites and hosts. We note that *k* in our model was defining the aggregation parameter that controls the extent of the over-dispersion of the worm population in the host, with a highly aggregated distribution when *k* < 1 and a more evenly distributed worm population for large *k* (i.e., *k* > 1) (77). In terms of influencing the elimination of worm burden in the community, this parameter has been shown to be less important (33, 78).

For the population-related parameters (*n*_*i*_ for *i* = *p, c, a*), only adult population (*n*_*a*_) was observed to be influential among all the hosts as well as infectious materials, but only for the case of *Ascaris lumbricoides* and *Trichuris trichiura*. This finding can be mainly attributed to the high adult population proportion considered in the model. Further, given that for a long time STH interventions have been focused toward school-going children and driven their prevalence to lower levels (48, 79), adults have now emerged as the new reservoir for the infections (80, 81), thus for elimination of STH (especially *Ascaris lumbricoides* and *Trichuris trichiura*) to be achieved, interventions should target adults (akin to CBD strategy), indeed our model already showed that this group greatly influenced the model outcome. In fact, Kenya has now began implementing, in pilot basis, the delivery of community-based mass treatment for STH infections, alongside the long-standing school-based MDA (37, 82, 83). This strategy aims to reduce the infection burden in the entire community, thus achieve the elusive STH elimination.

Similarly, transmission and fecundity-related parameters, β_*i*_ and λ_*i*_ for *i* = *p, c, a*, respectively, showed differing levels of influence on the model outcome relative to the specific host and worm species. The model analysis showed that infection transmission as well as environmental contamination was amplified by the pre-school children and adult population respectively. The high transmission rates in pre-school children could be due to their regular interaction with the contaminated environment (84). Whilst the high environmental contamination (fecundity) by adults can be explained by the density-dependence effects (adults proportion was high in the model), as well as the fact that adults currently harbor higher burden of the infections due to their low treatment coverage levels (79). If the high transmission rate among the pre-school children and high contamination rate by the adults are left un-addressed by the control programmes, then elimination period of STH might drag further.

## 5. Conclusion

This model sensitivity analysis demonstrated that for STH control programmes to effectively eliminate STH worm burden (and within a short period) in the entire community, key parameter groupings; combined effect of improved water source and sanitation, rounds of treatment offered, efficacy of the drug used during treatment, proportion of the adult population treated, mortality rate of the mature worms in the human host, and the strength of the density-dependence of worm egg production, need to be targeted and should be well coined within the package of interventions offered by the control programmes. This modeling results are significant to the Kenyan STH control program and to the global STH control community since it clearly Elucidate key parameters to be targeted for inclusion within the STH intervention packages, which is important information that is needed especially at this time when control programs globally are aiming to eliminate STH by the year 2030.

## Data Availability Statement

The original contributions presented in the study are included in the article/Supplementary Material, further inquiries can be directed to the corresponding author/s.

## Ethics Statement

The studies involving human participants were reviewed and approved by Kenya Medical Research Institute (KEMRI)'s Scientific and Ethics Review Unit (SSC Number 2206). Written informed consent to participate in this study was provided by the participants' legal guardian/next of kin.

## Author Contributions

CO conceptualized the study, formulated the model, developed the R codes and analyzed the models, and wrote the draft manuscript. CM provided the field data, interpreted the parasitological results, and reviewed the draft manuscript. NO, IO, and GM conceptualized the study, formulated the model, and reviewed the draft manuscript and provided overall scientific guidance. All authors participated in the interpretation of the findings, read and approved the final manuscript.

## Funding

This work was funded by GlaxosmithKline (GSK) Africa Non-Communicable Disease Open Lab through the DELTAS Africa Initiative Grant No. 107754/Z/15/Z-DELTAS Africa SSACAB. The DELTAS Africa Initiative is an independent funding scheme of the African Academy of Sciences (AAS)'s Alliance for Accelerating Excellence in Science in Africa (AESA) and supported by the New Partnership for Africa's Development Planning and Coordinating Agency (NEPAD Agency) with funding from the Wellcome Trust (Grant No. 107754/Z/15/Z) and the UK government. The funders had no role in the study design, data collection and analysis, decision to publish or preparation of the manuscript.

## Author Disclaimer

The views expressed in this publication are those of the author(s) and not necessarily those of GSK, AAS, NEPAD Agency, Wellcome Trust, or the UK government.

## Conflict of Interest

The authors declare that the research was conducted in the absence of any commercial or financial relationships that could be construed as a potential conflict of interest.

## Publisher's Note

All claims expressed in this article are solely those of the authors and do not necessarily represent those of their affiliated organizations, or those of the publisher, the editors and the reviewers. Any product that may be evaluated in this article, or claim that may be made by its manufacturer, is not guaranteed or endorsed by the publisher.
