# Supplementary material for: Sensitivity Analysis of a Transmission Interruption Model for the Soil-Transmitted Helminth Infections in Kenya
Source: Front Public Health. 2022 Mar 25;10:841883. doi: 10.3389/fpubh.2022.841883 (PMC8990131; doi:10.3389/fpubh.2022.841883)
Supplement: Supplementary file 1 [file Data_Sheet_1.pdf]

Supplementary Material

Ascaris lumbricoides

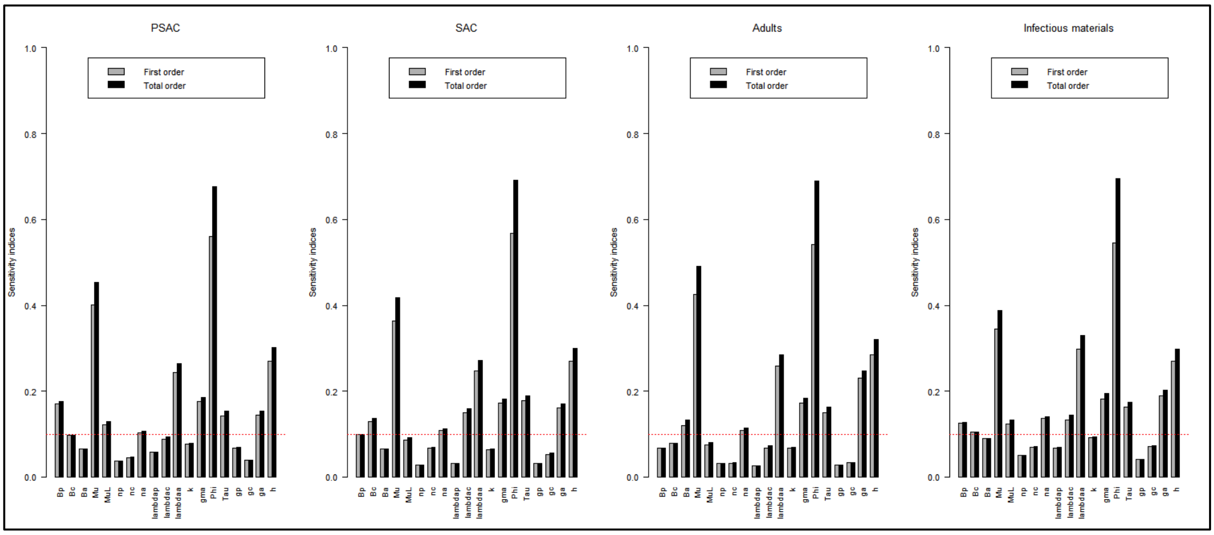

Figure S1: First and total-order sensitivity indices of the parameters using the eFAST method of sensitivity analysis for the case of *Ascaris lumbricoides*. The greater the sensitivity index, the more critical the parameter is to the model. The red dotted line indicated the cut-off sensitivity index value (SI=0.1) above which a parameter was deemed significantly influential to the model outcome.

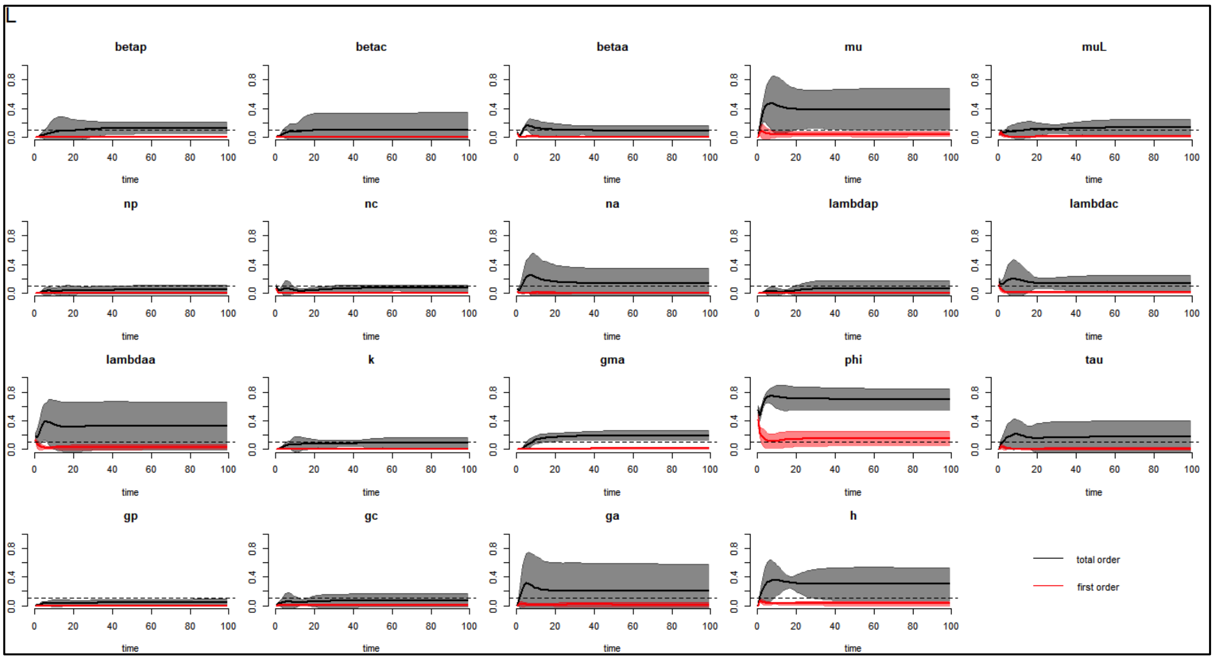

Figure S2: Plot of the first and total-order sensitivity indices of the parameters for infectious materials in the environment (i.e., eggs or larvae) for the case of *Ascaris lumbricoides*.

Hookworm

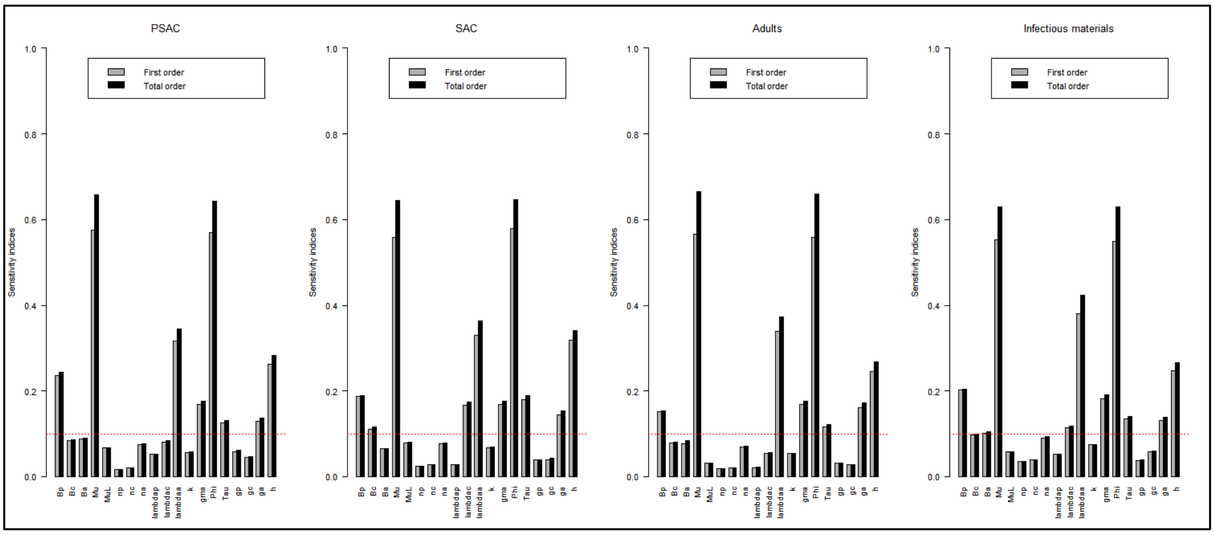

Figure S3: First and total-order sensitivity indices of the parameters using the eFAST method of sensitivity analysis for the case of hookworm. The greater the sensitivity index, the more critical the parameter is to the model. The red dotted line indicated the cut-off sensitivity index value (SI=0.1) above which a parameter was deemed significantly influential to the model outcome.

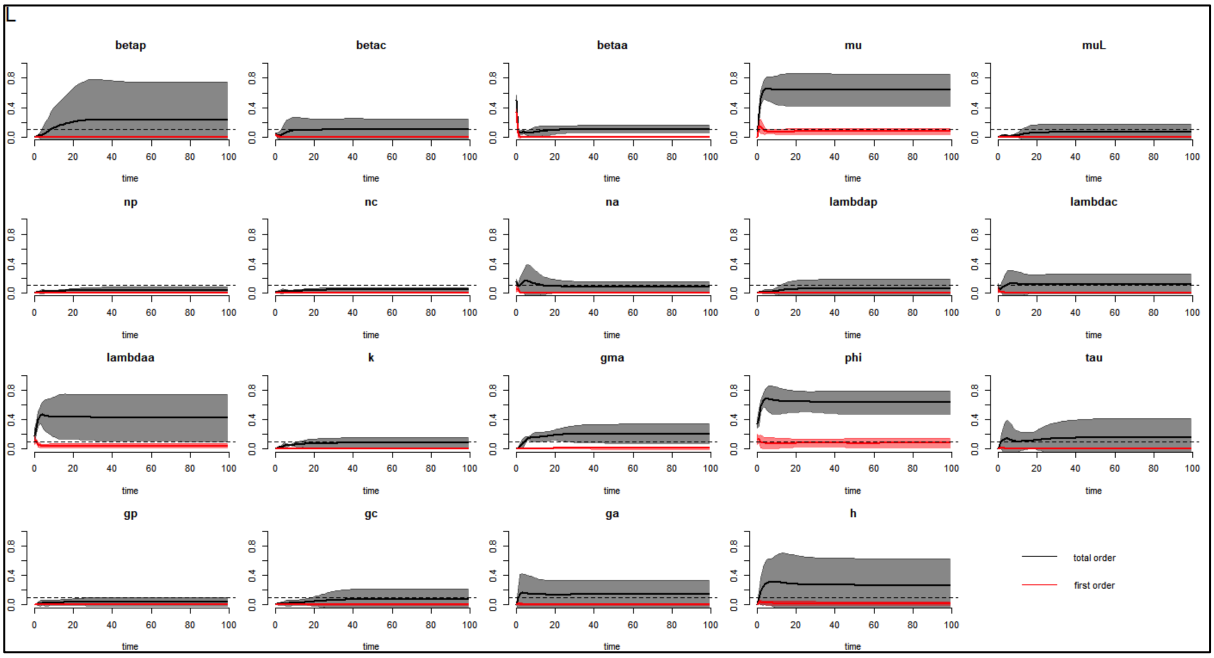

Figure S4: Plot of the first and total-order sensitivity indices of the parameters for infectious materials in the environment (i.e., eggs or larvae) for the case of hookworm.

Trichuris trichiura

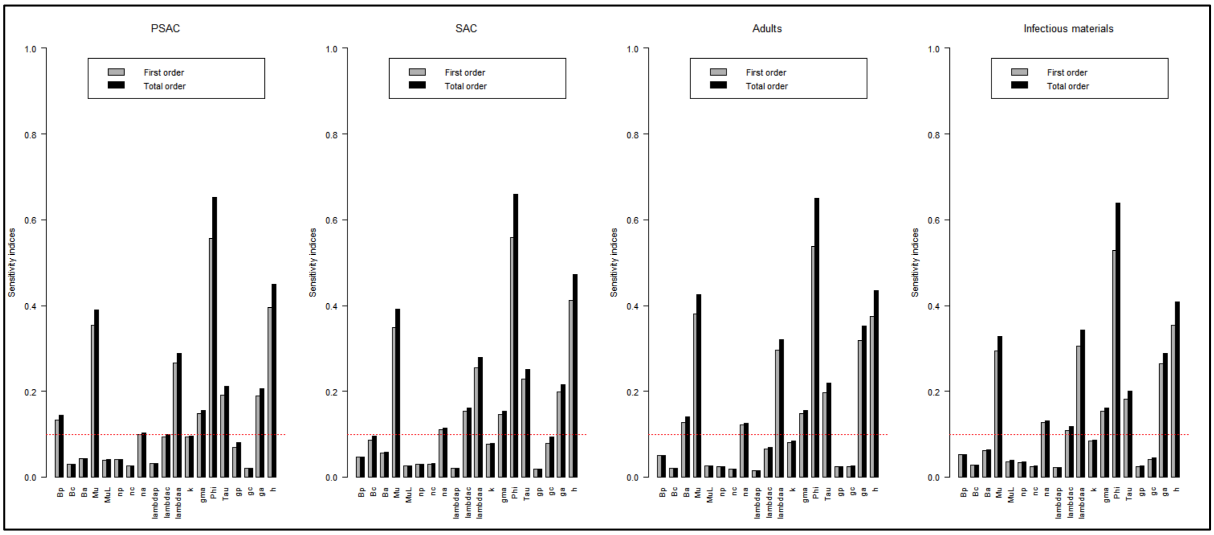

Figure S5: First and total-order sensitivity indices of the parameters using the eFAST method of sensitivity analysis for the case of *Trichuris trichiura*. The greater the sensitivity index, the more critical the parameter is to the model. The red dotted line indicated the cut-off sensitivity index value (SI=0.1) above which a parameter was deemed significantly influential to the model outcome.

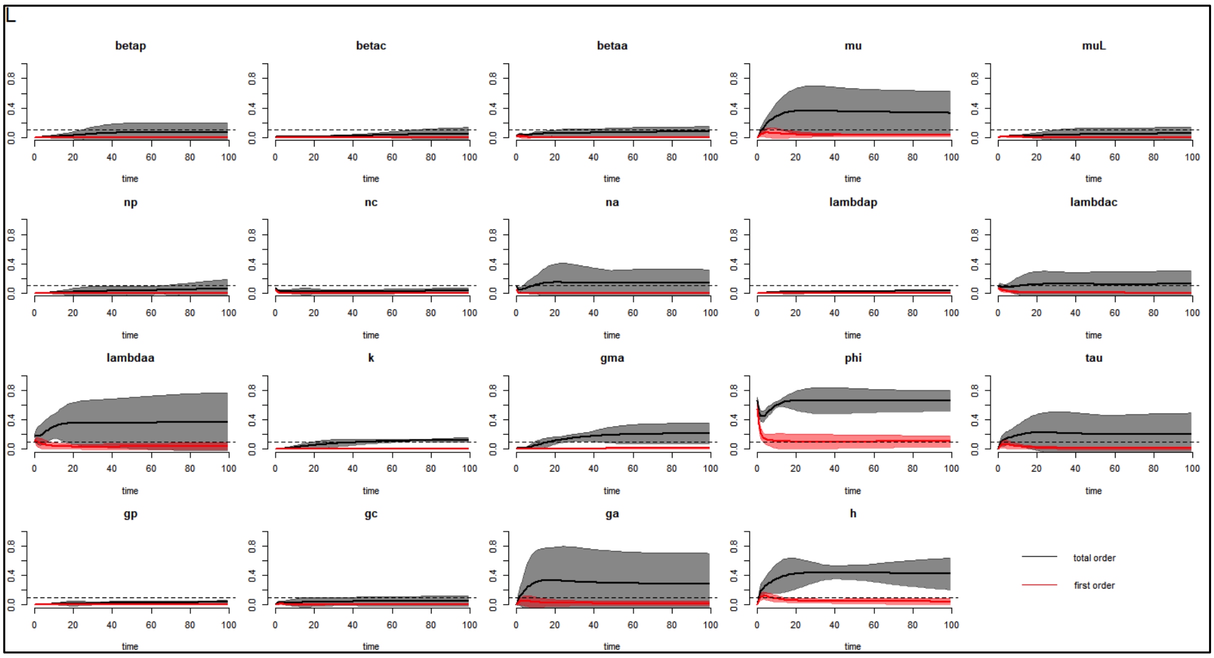

Figure S6: Plot of the first and total-order sensitivity indices of the parameters for infectious materials in the environment (i.e., eggs or larvae) for the case of *Trichuris trichiura*.
